# Repo-Man Coordinates Chromosomal Reorganization with Nuclear Envelope Reassembly during Mitotic Exit

**DOI:** 10.1016/j.devcel.2011.06.020

**Published:** 2011-08-16

**Authors:** Paola Vagnarelli, Susana Ribeiro, Lau Sennels, Luis Sanchez-Pulido, Flavia de Lima Alves, Toon Verheyen, David A. Kelly, Chris P. Ponting, Juri Rappsilber, William C. Earnshaw

**Affiliations:** 1 Wellcome Trust Centre for Cell Biology, Institute of Cell Biology, University of Edinburgh, Edinburgh EH9 3JR, UK; 2 MRC Functional Genomics Unit, Department of Physiology, Anatomy and Genetics, University of Oxford, Oxford OX1 3QX, UK

## Abstract

Repo-Man targets protein phosphatase 1 γ (PP1γ) to chromatin at anaphase onset and regulates chromosome structure during mitotic exit. Here, we show that a Repo-Man:PP1 complex forms in anaphase following dephosphorylation of Repo-Man. Upon activation, the complex localizes to chromosomes and causes the dephosphorylation of histone H3 (Thr3, Ser10, and Ser28). In anaphase, Repo-Man has both catalytic and structural functions that are mediated by two separate domains. A C-terminal domain localizes Repo-Man to bulk chromatin in early anaphase. There, it targets PP1 for the dephosphorylation of histone H3 and possibly other chromosomal substrates. An N-terminal domain localizes Repo-Man to the chromosome periphery later in anaphase. There, it is responsible for the recruitment of nuclear components such as Importin β and Nup153 in a PP1-independent manner. These observations identify Repo-Man as a key factor that coordinates chromatin remodeling and early events of nuclear envelope reformation during mitotic exit.

## Highlights

► Repo-Man/PP1 complex is activated in anaphase by Repo-Man dephosphorylation ► Repo-Man/PP1 dephosphorylates histone H3 at Thr3, Ser10, and Ser28 ► Repo-Man binds Importin β in anaphase and targets it to the chromosome periphery ► Repo-Man has both catalytic and structural functions during mitotic exit

## Introduction

Mitotic exit comprises a complex series of events that include sister chromatid segregation, mitotic spindle disassembly, nuclear envelope (NE) reformation, and chromosome decondensation. Many of these events are driven by inactivation of mitotic kinases and dephosphorylation of their substrates. For successful division, these disparate events require a strict temporal and spatial coordination (Güttinger et al., 2009).

In organisms with an open mitosis, NE reformation requires coordination between structural changes in chromatin and recruitment of nuclear pore complex (NPC) and membrane components to the surface of the segregating chromosomes. The process begins in late anaphase with the binding of NPC proteins to chromosomes. It is completed with the recruitment and fusion of membranes during telophase.

The coordination of chromatin decondensation with nuclear reassembly during mitotic exit is not well understood. One key step is dephosphorylation of proteins modified by CDKs and other mitotic kinases (De Wulf et al., 2009). Some of these phosphatases are constitutively active; however, recent studies have shown that activation of specific phosphatases is also required for mitotic exit (Queralt and Uhlmann, 2008; De Wulf et al., 2009).

During mitotic exit, protein phosphatase 1 (PP1) is involved in histone dephosphorylation (Hsu et al., 2000) and NE reassembly at the M/G1 transition (Steen et al., 2000). PP1 mutants in *Drosophila* show a mitotic delay with spindle organization defects, abnormal sister chromatid segregation, and excessive chromosome condensation (Axton et al., 1990; Chen et al., 2007). In *Drosophila*, reassembly of the NPC is blocked by the specific PP1/PP2A inhibitor okadaic acid (OA) (Onischenko et al., 2005). How this change in PP1 activity is regulated during anaphase is currently unknown.

Repo-Man was identified as a nuclear protein that is a specific regulatory subunit for PP1γ (Trinkle-Mulcahy et al., 2006). Repo-Man disperses in the cytoplasm as cells enter prophase but relocalizes abruptly to the chromatin at anaphase onset (AO). We previously found that CDK1-CyclinB can phosphorylate Repo-Man in vitro and that CDK inactivation by roscovitine causes the rapid relocalization of Repo-Man to the chromosomes. We identified the Repo-Man/PP1 complex as responsible for inactivation of a regulator of chromosome architecture (RCA) in anaphase (Vagnarelli et al., 2006). Thus, Repo-Man is a candidate factor involved in preparing mitotic chromatin for the transition to interphase.

Here we show that Repo-Man/PP1γ is indeed an anaphase-activated protein phosphatase that is regulated via Repo-Man phosphorylation. We also identified an unexpected role for Repo-Man as a factor that targets Importin β to chromatin during anaphase. This occurs via a direct interaction between the Repo-Man N-terminal region and Importin β that is negatively regulated by Repo-Man phosphorylation and does not require PP1 binding. Our studies thus reveal that Repo-Man has a dual role in nuclear reassembly during mitotic exit. It prepares the chromatin for decondensation by removing mitosis-specific chromatin marks and also targets early components of the reforming NE to the surface of the telophase chromosomes.

## Results

### CDK/Cyclin B Regulates Repo-Man Localization and PP1 Binding

**Figure 1:**
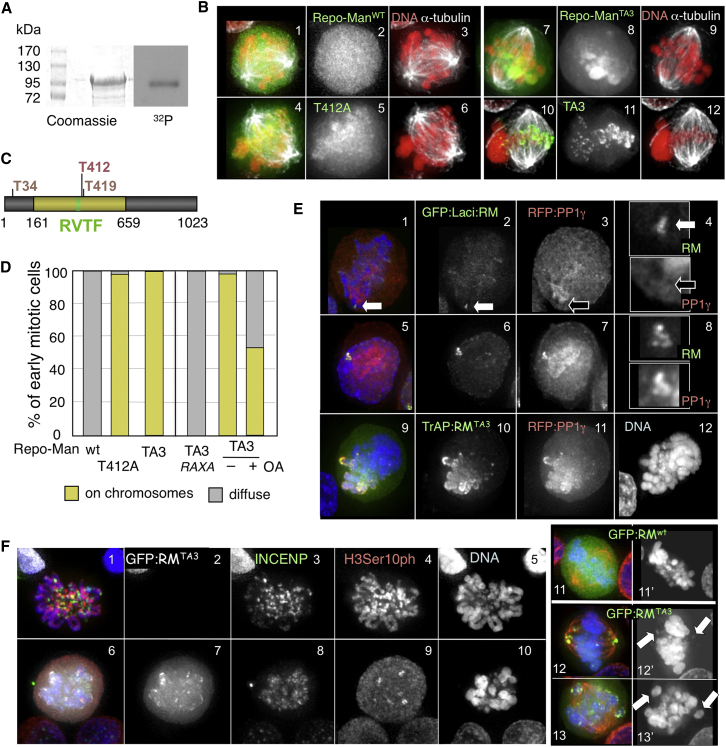
Repo-Man Phosphorylation Regulates the Localization and Activity of the Complex (A) Purified GST-Repo-Man^161–659^ was phosphorylated in vitro by cdc2/cyclinB. (B) Localization of GFP:Repo-Man and derivative mutants in prometaphase/metaphase. (1–3) Repo-Man^WT^, (4–6) Repo-Man^T412A^, (7–12) Repo-Man^TA3^; DNA (red), GFP:Repo-Man (green), alpha-tubulin (white). (C) Diagram of Repo-Man protein indicating (yellow) the region used for (A) and the sites where phospho null mutations were generated. (D) Quantitation of the experiments described in (B). OA, okadaic acid. For each condition n = 50. (E) DT40 cells carrying a LacO array integrated in a single chromosome site were transiently transfected with GFP:Laci-Repo-Man^WT^ (green) and RFP-PP1γ (red). Repo-Man and PP1 do not colocalize in metaphase (1–4) but do colocalize in interphase (5–8). (9–12) TrAP:Repo-Man^TA3^ (stained green with anti-Repo-Man antibody) recruits RFP-PP1γ (red) to chromosomes in metaphase. (F) DT40 cells transfected with Repo-Man^TA3^ mutant show decreased levels of H3Ser10ph, diffuse localization of INCENP, and chromosome alignment defects. Repo-Man^TA3^ (white), INCENP (green), H3Ser10ph (red), DNA (blue). (1–5) untransfected cell; (6–10) transfected cell. (11–13) Cell transfected with GFP:Repo-Man^WT^ (11) or Repo-Man^TA3^ (12 and 13). Repo-Man (green), alpha-tubulin (red), and DNA (blue).

Repo-Man is phosphorylated during mitosis (Cantin et al., 2008; Dephoure et al., 2008; Malik et al., 2009; Mayya et al., 2009; Olsen et al., 2010), and we previously showed that the protein can be phosphorylated in vitro by CDK1-Cyclin B (Vagnarelli et al., 2006). We used mass spectrometry on a bacterially expressed Repo-Man fragment encompassing the PP1-binding domain after in vitro phosphorylation with CDK1-cyclin B to map potential phosphorylation sites and determine their consequence for the formation and regulation of the complex (Figure 1A). This analysis identified threonine 412 (T412) as the main residue phosphorylated by CDK1-cyclin B in vitro. Indeed, phosphorylation of this residue, together with others, was reported in a mass spectrometry atlas of mitotic phosphoproteins (Dephoure et al., 2008; Mayya et al., 2009).

Phosphorylation of T412 regulates Repo-Man localization in early mitosis. When this site is mutated to alanine, a fraction of Repo-Man^T412A^ binds prematurely to chromosomes while cells are still in prometaphase/metaphase (Figures 1B, 4–6, and 1D). To test whether other phosphorylated sites contribute to Repo-Man localization, we mutated two other conserved putative CDK sites to A: one near the N terminus (T34A), and the other close to T412 (T419A) (Figure 1C). This triple mutant (referred to as Repo-Man^TA3^) shows a more prominent localization to the chromosomes in prometaphase (Figures 1B, 7–12, and 1D). Importantly, the localization of Repo-Man^TA3^ and Repo-Man^T412A^ is indistinguishable from wild-type Repo-Man after AO.

For some PP1 regulatory subunits, it has been demonstrated that phosphorylation of residues proximal to or within the PP1-binding sites can influence complex formation (Terrak et al., 2004; Kim et al., 2010; Liu et al., 2010). We therefore established a system to test whether Repo-Man binding to PP1 is regulated by phosphorylation in vivo in early mitosis.

Repo-Man fused to GFP:Laci recruits RFP-PP1γ to an array of LacO repeats at a single chromosomal locus in interphase DT40 cells (Figure 1E, 5–8). However, we could not detect RFP-PP1γ colocalization with GFP:Laci-Repo-Man in prometaphase/metaphase (Figure 1E, 1–4). These findings suggest that dephosphorylation of residues around the RVTF motif (PP1-binding site) may be required for PP1 binding to Repo-Man. Indeed, Repo-Man^TA3^ does recruit RFP-PP1γ to chromosomes in early mitosis (Figure 1E, 9–12).

We hypothesized that bound PP1γ might dephosphorylate other sites on Repo-Man and that this might be required to allow Repo-Man to relocalize to anaphase chromosomes. To test this hypothesis, we generated a Repo-Man^TA3^ mutant where the PP1-binding site was mutated to RAXA (Repo-Man^TA3RAXA^). This blocks PP1 binding (Trinkle-Mulcahy et al., 2006). This mutant did not localize on the chromosomes in prometaphase-metaphase (Figure 1D). Moreover, inhibition of PP1/PP2A activity by OA also reduced the chromosomal localization of the Repo-Man^TA3^ mutant (Figure 1D).

This phosphoregulation of Repo-Man:PP1 in mitosis is functionally significant. Recent findings revealed H3Thr3 phosphorylation to be essential for localization of the chromosomal passenger complex (CPC) to centromeres in mitosis (Kelly et al., 2010; Wang et al., 2010; Yamagishi et al., 2010). Therefore, premature localization of Repo-Man:PP1 to chromosomes would be expected to interfere with CPC function. Indeed, INCENP was diffuse on the chromosomes in cells expressing Repo-Man^TA3^ (Figure 1F, 6–10). Also, nonaligned chromosomes were observed in transfected cells with a bipolar spindle (Figure 1F, 12–13). Similar results have been obtained in another recent study (Qian et al., 2011).

These experiments reveal that Repo-Man phosphorylation both promotes Repo-Man dissociation from the chromatin until AO and ensures that Repo-Man cannot bind PP1 and target it to substrates in early mitosis. Thus, Repo-Man/PP1 phosphatase holoenzyme is specifically activated on chromatin at AO.

### Identification of Repo-Man-Binding Partners in Anaphase

**Figure 2:**
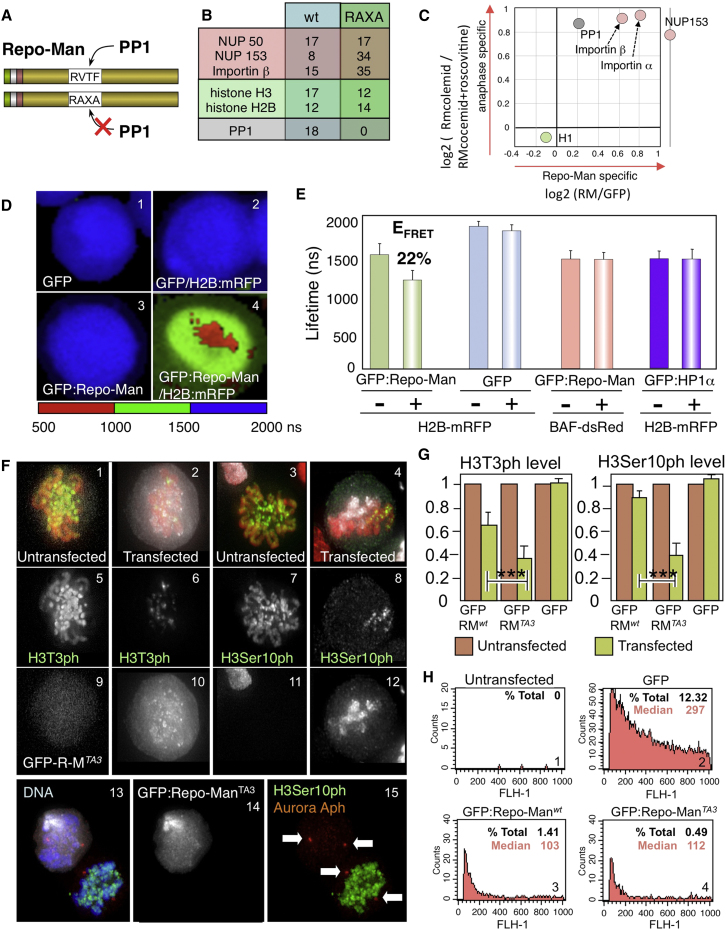
Repo-Man Interacts with Histones and Nuclear Pore Complex Components (A) Diagrams of TrAP:tagged Repo-Man^WT^ and Repo-Man^RAXA^ (PP1-nonbinding mutant). (B) Proteins identified by mass spectrometry in the indicated pull-down experiments. (C) Summary of SILAC results; the log_2_ ratio between Repo-Man plus roscovitine (anaphase) and Repo-Man minus roscovitine (prometaphase) is plotted against the log_2_ ratio between Repo-Man and GFP (control)—both from roscovitine-treated cells. Nup153 was only observed in the cell cycle experiment (y axis). (D and E) FLIM/FRET analysis for H2B:mRFP and GFP:Repo-Man. (D) Color-coded representation of the lifetime for GFP:Repo-Man or GFP alone (donors) in the absence or presence of H2B:mRFP (acceptor). (E) Histogram of the lifetime values measured in the different experiments. Error bars, average ± SD. (F and G) Repo-Man premature localization to the chromosomes causes dephosphorylation of histone H3. DT40 cells were transfected with Repo-Man^WT^, Repo-Man^TA3^, or GFP alone. Twenty-four hours later, cells were fixed and stained with the indicated antibodies. (F) Prometaphases of untransfected (1, 5, 9; 3, 7, 11) or transfected (2, 6, 10; 4, 8, 12) cells stained with antibody (green) to anti-H3T3ph (1, 2, 5, 6) or H3Ser10ph (3, 4, 7, 8); GFP:Repo-Man^TA3^ (white) and DNA (red). (13–15) Cells were stained with antibody to H3Ser10ph (green) and anti-phospho-Aurora A (red); DNA (blue); Repo-Man^TA3^ (white). Arrows indicate the phospho-Aurora A staining. (G) Quantitation of the staining from the experiments in (F). Error bars, average ± SD. ^∗∗∗^p < 0.001. (H) FACS analyses of DT40 cells transfected with the indicated constructs. The x axis depicts the GFP fluorescence of the transfected population. % Total, the percentage of transfected cells; Median, median expression level.

In order to isolate Repo-Man interactors during mitotic exit, DT40 cells stably expressing TrAP-tagged (Samejima et al., 2008) hRepo-Man^WT^ or PP1-nonbinding mutant hRepoMan^RAXA^ (Figure 2A; see also Figure S1C available online) were blocked in mitosis with colcemid, and then forced to exit from mitosis by addition of the CDK inhibitor roscovitine. We previously showed that hRepo-Man can recognize physiologically relevant targets in chicken cells during mitotic exit because overexpression of hRepo-Man^RAXA^ can rescue anaphase chromatid segregation in DT40 cells conditionally lacking condensin (Vagnarelli et al., 2006).

DT40 cultures treated with colcemid overnight had a mitotic index of 70%–85% with Repo-Man diffuse in the cytoplasm. Addition of roscovitine for 15′ caused a rapid relocalization of Repo-Man to the chromatin (Vagnarelli et al., 2006). Proteins solubilized from the chromosome fraction were purified using streptavidin beads and an S-protein column (Figure S1D), then analyzed by mass spectrometry.

Repo-Man^WT^, but not Repo-Man^RAXA^, pulled down PP1γ and β (Figure 2B). Among other Repo-Man-associated proteins, the highest number of peptides was obtained from histones, Importin β, and NPC components NUP50 and NUP153.

A second set of pull-down experiments used SILAC to identify Repo-Man interactors whose binding was enriched in anaphase versus prometaphase. We also assessed specificity by comparing Repo-Man-expressing cells with a cell line expressing only TrAP:GFP (Figure S1E). The results were plotted as a two-dimensional diagram, on which anaphase-specific Repo-Man-interacting proteins appear in the upper right quadrant (Figure 2C). There, we found PP1γ and β, Importin α, and Importin β. NUP153 was enriched in the anaphase fraction, but not identified in the other pull-down experiment. This analysis confirmed that the interaction of Repo-Man with PP1 is more stable in anaphase and requires dephosphorylated Repo-Man (Figure 1E and Figure S1B).

These experiments thus revealed interactions of Repo-Man with Importin β and NUP153 during anaphase.

### Repo-Man Binds Chromatin in Close Proximity to H2B and Directs Histone H3 Dephosphorylation

**Figure 3:**
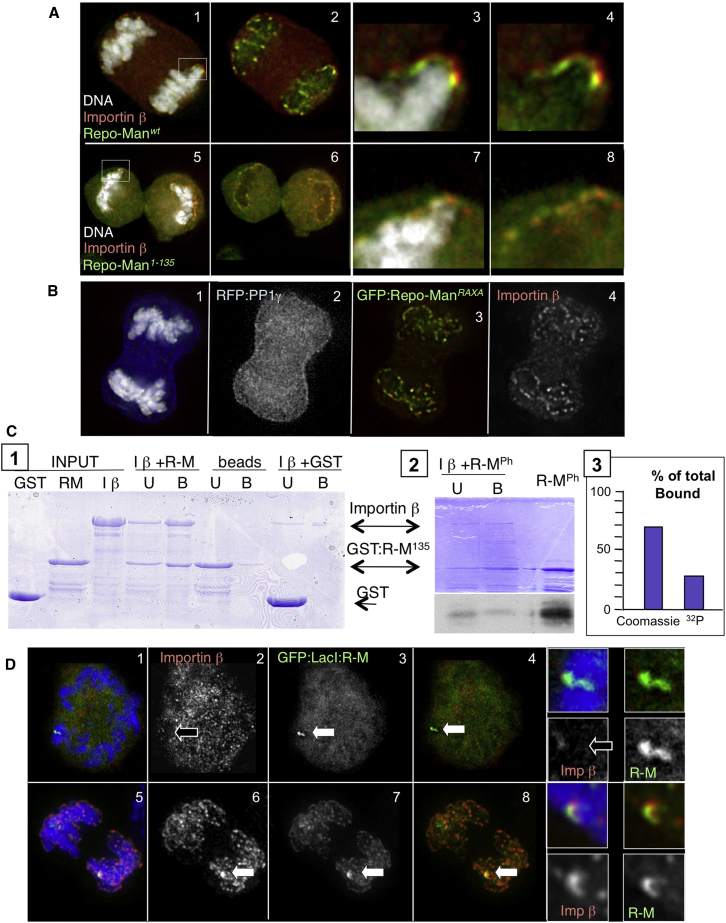
Repo-Man Binds Importin β, and the Binding Is Regulated by Repo-Man Phosphorylation (A) Anaphase DT40 cells were transfected with GFP:Repo-Man^WT^ (1–4) or Repo-Man^1–135^ (5–8) (green) and stained with an antibody recognizing the endogenous Importin β (red). DNA is white. (B) Anaphase cells transfected with GFP:Repo-Man^RAXA^ (PP1-nonbinding mutant) (green), RFP-PP1γ (blue), and stained for Importin β (red); DNA is white. (C) in vitro binding of Repo-Man^1–135^ and Importin β. (1) GST, GST-Repo-Man^1–135^ purified from *E*. *coli*, incubated with His-Importin β, and captured with Ni beads. (2) GST-Repo-Man was in vitro phosphorylated with CDK1-Cyclin B before performing the binding experiment as in (1). Upper, Coomassie; lower, autoradiograph. (3) Quantitation of the amount of Repo-Man in the pellet fraction from the Coomassie and from the autoradiograph (^32^P). U, unbound fraction; B, bound fraction. (D) DT40 cells carrying a LacO array integrated in a single locus were transiently transfected with GFP:Laci-Repo-Man (green) and then stained for Importin β (red). (1–4) Prometaphase cells show no colocalization between GFP:Laci-Repo-Man and Importin β. (5–8) In anaphase cells GFP:Laci-Repo-Man (green) and Importin β (red) colocalize at the integration site (white arrows).

**Figure 4:**
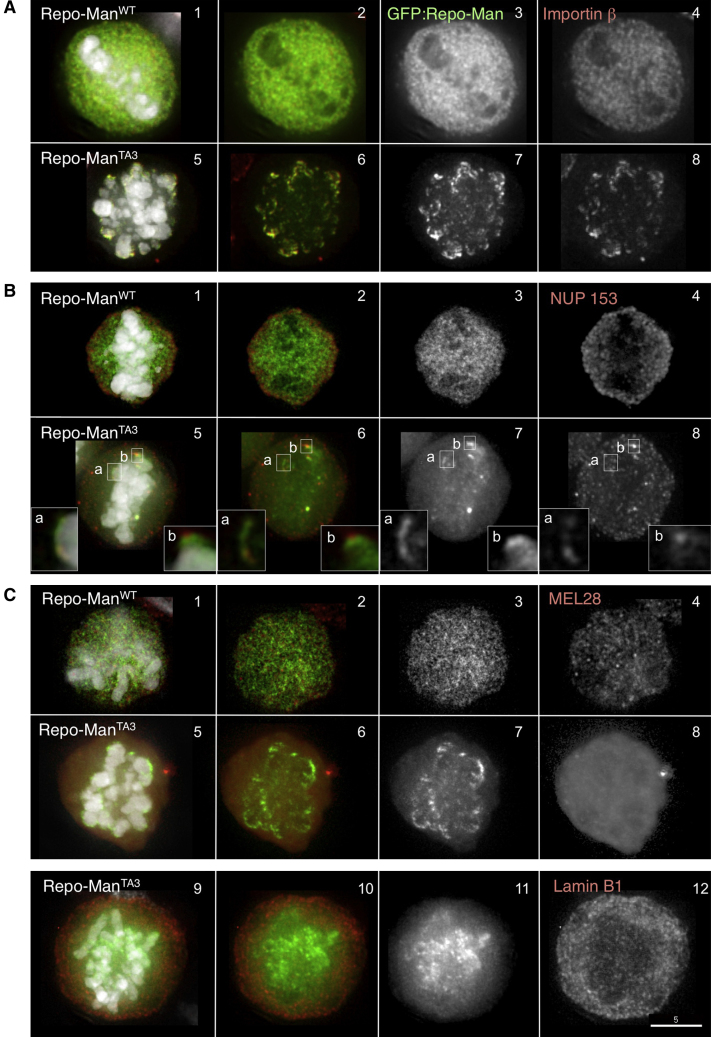
Repo-Man^TA3^ Is Sufficient to Trigger Deposition of Importin β and Nup153 on the Chromosomes Independently of Mitotic Exit (A and B) DT40 cells transfected with GFP:Repo-Man^WT^ (A and B, 1–4) or GFP:Repo-Man^TA3^ (green) (A and B, 5–8) and stained (red) for Importin β (A) or Nup153. Insets in (B) show a blowup of the indicated regions of enrichment for Repo-Man and Nup153. (C) DT40 cells transfected with GFP:Repo-Man^WT^ (green) (1–4) or GFP:Repo-Man^TA3^ (green) (5–12) and stained with antibodies (red) against MEL-28 (1–8) or LaminB1 (9–12).

Our pull-down results suggest that Repo-Man is a chromatin-associated protein, as shown recently for *Xenopus* Repo-Man (Peng et al., 2010). A FLIM-FRET approach confirmed that Repo-Man binds close to H2B on chromatin. We observed a significant fluorescence lifetime decrease for GFP:Repo-Man in the presence of H2B:mRFP (Figures 2D, 3 and 4, and 2E), but no effect on the lifetime of the control GFP (Figures 2D, 1 and 2, and 2E). This decrease corresponds to an E_FRET_ of 22% for Repo-Man. Therefore, Repo-Man and H2B are in close proximity during interphase in vivo. In other control experiments we detected no significant FRET between GFP:Repo-Man and the chromatin protein dsRed-BAF or between GFP:HP1α and H2B:mRFP (Figure 2E).

PP1 is believed to be responsible for H3S10 dephosphorylation in anaphase (Hsu et al., 2000); however, the relevant PP1-targeting subunit is not known. To test whether Repo-Man/PP1 can dephosphorylate the major mitotic phospho-sites on histone H3 (Thr3, Ser10, and Ser28), we exploited the Repo-Man^TA3^ mutant that localizes to chromosomes and binds PP1 in prometaphase.

Targeting Repo-Man^TA3^ to prometaphase chromosomes in transient transfections decreased H3 phosphorylation at Ser10, Thr3 (Figures 2F and 2G), and Ser28 (data not shown). Overexpressed wild-type Repo-Man also lowered H3 phosphorylation at T3 and S10, even though the protein does not localize stably to chromosomes. However, expression of the mutant protein at a similar level (Figure 2H) caused a significantly greater decrease in H3 phosphorylation (p < 0.001 for both S10 and T3).

The activity of Repo-Man^TA3^/PP1 observed in this experiment does not reflect general nonspecific dephosphorylation of mitotic phosphoproteins. The mitotic phosphorylation of Aurora A (Bayliss et al., 2003; Eyers et al., 2003; Hirota et al., 2003) was maintained in mitotic cells transfected with Repo-Man^TA3^ even when H3Ser10 phosphorylation was lost (Figure 2F, 13–15). Moreover, Repo-Man depletion by RNAi caused a persistence of H3Ser10ph and H3Thr3ph in postmitotic cells (see below).

We conclude that Repo-Man binds to nucleosomes and directs PP1 to dephosphorylate histone H3 at the three major mitotic phospho-sites.

### Repo-Man Binds Importin β and Targets It to Anaphase Chromosomes

Consistent with the specific interaction between Repo-Man and Importin β observed in anaphase cell lysates (Figure 2C), GFP:Repo-Man^WT^ and Importin β partially colocalize at the periphery of anaphase chromosomes in cells (Figure 3A, 1–4). To determine whether this was due to direct binding, we first identified a minimal fragment of Repo-Man (residues 1–135 from the Repo-Man N terminus) that colocalizes with Importin β on anaphase chromosomes (Figure 3A, 5–8). Both full-length Repo-Man (data not shown) and GST-Repo-Man^1–135^ expressed in bacteria bind directly to His-Importin β in vitro (Figure 3C, 1). Importantly, this binding is independent of Repo-Man's function as a PP1-targeting subunit. Repo-Man^1–135^ lacks the RVTF motif and cannot bind PP1. Furthermore, full-length Repo-Man^RAXA^ mutant, which cannot bind PP1 (Trinkle-Mulcahy et al., 2006) and has a dominant-negative effect displacing PP1 from anaphase chromatin (Figure 3B, 2), also colocalizes with Importin β at the chromosome periphery (Figure 3B, 3 and 4).

Both Repo-Man and Importin β are diffuse in prometaphase/metaphase, so to confirm their interaction in vivo, we fused Repo-Man to GFP:Laci in order to tether it at a discrete site. This Laci fusion construct was expressed in DT40 cells carrying a LacO array integrated at a single locus. As predicted by our proteomic analysis, Importin β was highly enriched at sites of GFP:Laci-Repo-Man binding in anaphase cells (Figure 3D, 5–8) but was not detected at GFP:Laci-Repo-Man foci in prometaphase/metaphase cells (Figure 3D, 1–4). Similar results were obtained when the N-terminal fragment GFP:Laci-Repo-Man^1–135^ was expressed in cells carrying a LacO integration on a single chromosome. GFP:Laci-Repo-Man^1–135^ colocalized with Importin β in telophase cells (Figure S2A, 5–8), but never in prometaphase/metaphase cells (Figure S2A, 1–4).

These results suggest that the Repo-Man binding to Importin β is regulated in a cell cycle-specific manner. Repo-Man has several putative CDK phosphorylation sites within its N-terminal regions, a number of which are phosphorylated in vivo (Cantin et al., 2008; Dephoure et al., 2008; Malik et al., 2009; Olsen et al., 2010) (Figure S1A). Indeed, phosphorylation of purified GST-Repo-Man^1–135^ by purified cdc2/cyclinB reduced its binding in vitro to Importin β by 50% (Figure 3C, 2 and 3). Consistent with this, premature localization of the Repo-Man^T412A^ or Repo-Man^TA3^ phospho-site mutants was sufficient to target both Importin β (Figure 4A, 5–8) and Nup153 (Figure 4B, 5–8) to chromosomes before AO. This premature targeting was not seen with wild-type Repo-Man (Figures 4A, 1–4, and 4B, 1–4).

The results thus far reveal that Repo-Man binds directly to Importin β independent of its binding to PP1 and that the binding is negatively regulated by CDK phosphorylation.

### Repo-Man Has Two Independent Chromatin-Targeting Domains

**Figure 5:**
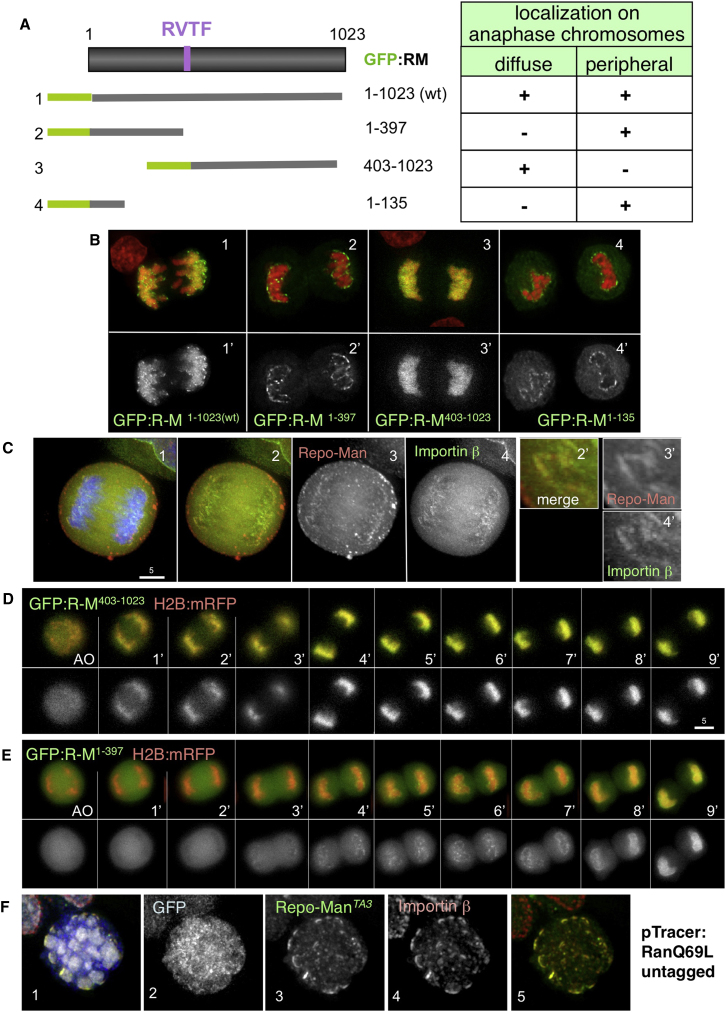
Repo-Man Contains Two Independent Chromosome-Targeting Domains (A) Diagram of Repo-Man showing the deletion mutants generated (1–4) and their localization in anaphase. (B) Anaphase cells transfected with GFP:Repo-Man^1–1203(WT)^ (1), GFP:Repo-Man^1–397^ (2), GFP:Repo-Man^403–1023^ (3), and GFP:Repo-Man^1–135^ (4). (C) Untransfected HeLa anaphase cell stained with an antibody recognizing endogenous Repo-Man (red) and Importin β (green); 2′–4′ blowups showing the colocalization of the two proteins. (D and E) Stills from movies of cells transfected with H2B:mRFP (red) and GFP:Repo-Man^403–1023^ (D) or GFP:Repo-Man^1–397^ (E) (green). AO, A onset. Lower panels show the GFP channel. (F) Mitotic cell transfected with TrAP:Repo-Man^TA3^ (green) plus RanQ69L in pTracer (GFP, blue) and stained with antibodies against Importin β (red).

Importin β, NUP50, and NUP153 are all recruited to chromatin very early during anaphase (Dultz et al., 2008). At this time, most Repo-Man localizes diffusely on the chromatin, although careful inspection also reveals the protein in foci that overlap with Importin β at the chromosome periphery (Figure 5B, 1). This colocalization was also observed in anaphase human cells using specific antibody to detect endogenous Repo-Man (Figure 5C).

How can a protein that localizes to bulk chromatin in anaphase and directs the global dephosphorylation of histone H3 also function in recruiting Importin β to the periphery of anaphase chromosomes? In order to gain insight into the underlying mechanism, we assessed the localization of several truncated forms of Repo-Man in anaphase DT40 cells (Figures 5A and 5B).

This analysis revealed that Repo-Man has two independent anaphase-specific chromosome-targeting domains. C-terminal Repo-Man^403–1023^, which excludes the PP1-binding RVTF motif (Figure 5A), is diffuse in metaphase but then localizes homogeneously on anaphase chromatin without enrichment at the periphery (Figure 5B, 3). All further truncations of this region of the protein failed to localize to chromatin in anaphase (data not shown). N-terminal Repo-Man^1–397^ (which also excludes the RVTF motif) is also diffuse in metaphase but localizes to foci at the chromosome periphery in anaphase (Figure 5B, 2). A further truncation, Repo-Man^1–135^, also targets to the chromosome periphery (Figure 5B, 4) and colocalizes with Importin β during anaphase (Figure 3A, 5–8). Importantly, the same localization was observed after expressing N-terminal and C-terminal Repo-Man in HeLa cells (Figure S3A).

How can a single protein have two different targeting motifs that function at the same cell cycle stage? Indeed, the function of these motifs is temporally resolved during mitotic exit. We transiently transfected constructs expressing GFP:Repo-Man^1–397^ or GFP:Repo-Man^403–1023^ together with H2B:mRFP into DT40 cells and imaged cells every minute starting during prometaphase. These analyses showed that Repo-Man^403–1023^ targets to the chromatin within 1 min after AO, reaching a maximum intensity between 3 and 4 min (Figure 5D). In contrast, Repo-Man^1–397^ starts accumulating at the periphery of the chromosomes only later during mitotic exit (3 min after AO) (Figure 5E), but before the appearance of Lamin A (Figure S3E). Its recruitment increases during telophase and is completed in the reforming nuclei (Figure 5E and Movies S1 and S2).

Both N-terminal and C-terminal Repo-Man are nuclear in interphase. Three observations argue against the trivial hypothesis that the colocalization of Repo-Man with Importin β in anaphase simply reflects ongoing nuclear import. First, live cell analysis reveals that the two proteins colocalize on anaphase chromosomes 4 min prior to detectable nuclear import (Figure S2B). Second, a GFP:Laci-SV40-NLS fusion protein does not recruit Importin β in telophase even when it is highly concentrated at a chromosomal LacO array (Figure S2A, 9–16). Third, overexpression of the active Ran mutant Q69L, which should promote release of import substrates from Importin β, has no detectable effect on the colocalization of Importin β and Repo-Man^TA3^ in early mitosis (Figure 5F and Figure S2C).

These results reveal that Repo-Man has two chromosome-targeting domains that function in a spatiotemporally independent manner during mitotic exit.

### Repo-Man Participates in Both Chromatin Remodeling and Nuclear Envelope Reassembly in Anaphase

**Figure 6:**
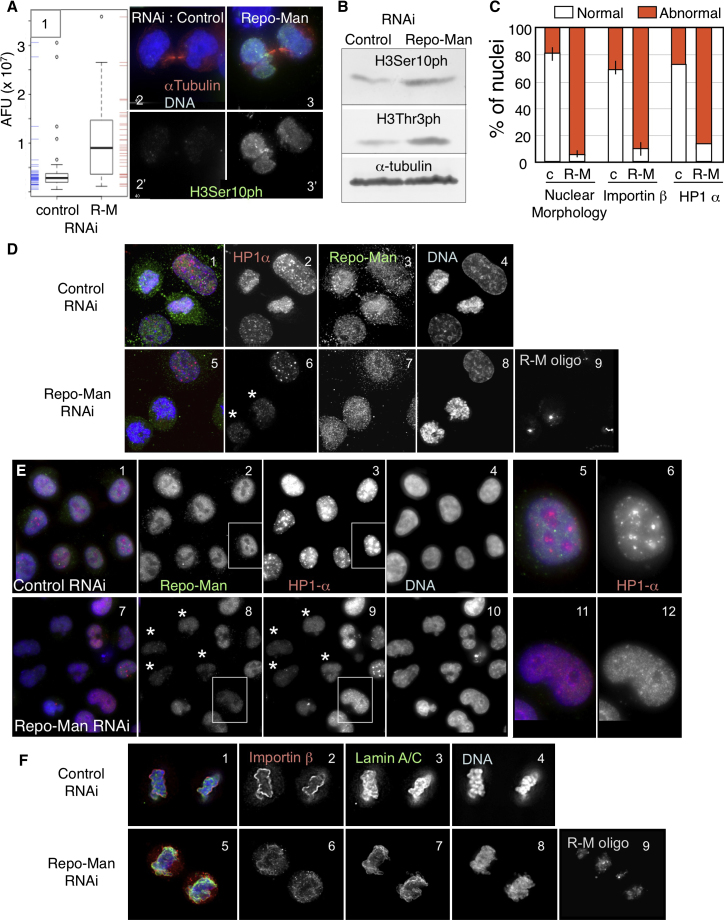
Repo-Man Depletion Affects H3 Dephosphorylation and HP1 Loading during Mitotic Exit (A and B) H3 phosphorylation in cells after control and Repo-Man RNAi. (A) (1) Quantitation of H3Ser10ph levels in postmitotic cells still joined by midbodies after control or Repo-Man RNAi (R-M). (2 and 3) Postmitotic cells from control (2 and 2′) or Repo-Man RNAi (3 and 3′) stained for α-tubulin (red), H3Ser10Ph (green), and DNA (blue). (B) Whole-cell extracts from control or Repo-Man RNAi were blotted with antibodies to H3Ser10, H3Thr3, and α-tubulin. (C) Quantification of the phenotypes of Repo-Man RNAi. c, control RNAi; R-M, Repo-Man RNAi. Error bars, average ± SD. (D) Telophase/cytokinesis from HeLa cells transfected with control (1–4) or Repo-Man oligos (5–9) and stained 48 hr later for HP1α and Repo-Man. (E) HeLa cells transfected with control (1–6) or Repo-Man oligos (7–12) and stained for Repo-Man and HP1α (1–12). (5 and 6) and (11 and 12) show blowups of boxed areas in 2 and 8. (F) Telophase/cytokinesis from HeLa cells transfected with control (1–4) or Repo-Man oligos (5–9) and stained for Importin β and Lamin A/C.

RNAi experiments in HeLa cells confirmed that Repo-Man targets PP1 for dephosphorylation of histone H3 during mitotic exit. These experiments used an oligonucleotide previously shown to be effective at specifically knocking down human Repo-Man (Trinkle-Mulcahy et al., 2006). When pairs of cells undergoing mitotic exit (e.g., still joined by an intercellular bridge) were examined following Repo-Man RNAi, we observed significantly elevated levels of histone H3 phosphorylated at Ser10 (Figure 6A). After Repo-Man depletion, the bulk cell population had a 2.9× higher level of H3Ser10ph and 3.2× higher level of H3Thr3ph relative to the control RNAi, despite the fact that the mitotic index is, if anything, slightly lower following Repo-Man RNAi (control RNAi, 3%; Repo-Man RNAi, 1.5%; Figure 6B).

In *Neurospora*, PP1 depletion causes a decrease in the levels of H3K9me3 (Adhvaryu and Selker, 2008), the histone mark that directs HP1 binding to chromatin (Bannister et al., 2001; Lachner et al., 2001). HP1 binding to this mark has been reported to be negatively regulated by phosphorylation of H3S10 in early mitosis (Fischle et al., 2005; Hirota et al., 2005). We therefore examined the localization of HP1α in Repo-Man-depleted cells undergoing mitotic exit.

HP1α normally accumulates on chromosomes during mitotic exit (Figure 6D, 1–4, and Figure S4A). This accumulation was substantially decreased following Repo-Man RNAi (Figure 6D, 5–9). Nuclear staining for HP1α remained low also in interphase following Repo-Man depletion. Furthermore, the typical accumulation of HP1 in heterochromatic nuclear foci was absent (Figure 6E, 5, 6, 11, and 12).

Surprisingly, Repo-Man depletion also caused dramatic changes in nuclear morphology after mitosis. This phenotype appears to result from depletion of the N-terminal Repo-Man module that targets Importin β to the chromosome periphery during mitotic exit.

Importin β targeting to the chromosome periphery normally occurs in anaphase prior to lamina deposition (Figures S4B and S4C). However, in Repo-Man-depleted cells, Importin β was not yet properly loaded around the chromatin in late mitotic figures when robust lamina staining was already present (Figure 6F, 5–9).

**Figure 7:**
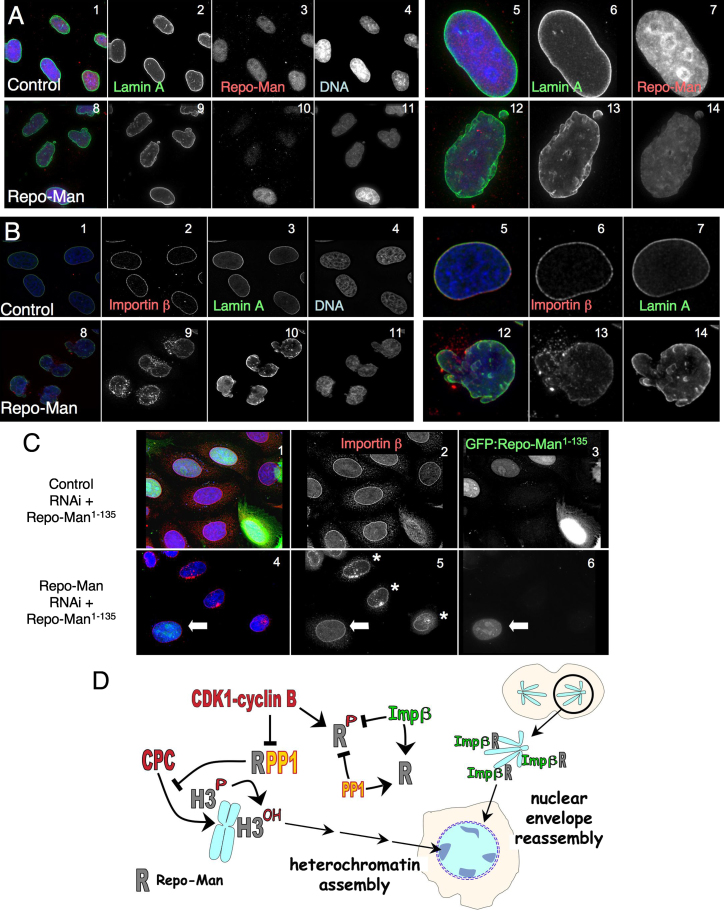
Repo-Man N-Terminal Domain Has a Structural Function during Mitotic Exit (A and B) HeLa cells transfected with control (A, 1–7, and B, 1–7) or Repo-Man oligos (A, 8–14, and B, 8–14). Forty-eight hours later, cells were fixed and stained for Lamin A/C and Repo-Man (A, 1–14); or Lamin A/C and Importin β (B, 1–14). Panels 5–7 and 12–14 show blowups of selected nuclei. (C) HeLa cells were transfected with control or Repo-Man oligos plus plasmid expressing GFP:Repo-Man^1–135^ and stained 48 hr later for Importin β. The nucleus of the cell transfected with GFP:Repo-Man^1–135^ (white arrow) has a normal distribution of Importin β and an improved nuclear morphology, compared to nearby untransfected cells (asterisks). (D) Model for the activation and function of the Repo-Man/PP1 complex during mitotic exit.

Strikingly, in HeLa cells depleted of Repo-Man by RNAi, lamin A/C staining revealed clearly abnormal nuclear shapes (Figures 7A and 7B and Figure S5A). In cells showing this abnormal nuclear shape, Importin β accumulated in patches in the cytoplasm that resembled annulate lamellae (Figure 7B) (Ito et al., 2007). Indeed, staining of Repo-Man-depleted cells with the mAb414 antibody recognizing multiple nucleoporins revealed the same cytoplasmic patches appearing after Repo-Man RNAi (Figure S5B).

Repo-Man overexpression also caused nuclei to exhibit an abnormal morphology. Overexpressed Repo-Man often localized in patches in the cytoplasm (Figure S6, 1–5). In those cells, Importin β was delocalized from the nuclear rim and accumulated in the same patches. Repo-Man overexpression also caused a reduction of NUP153 present at the NE (Figure S6, 6–10). However, under these conditions, staining for both ELYS/MEL-28, one of the earliest known proteins deposited on chromatin in anaphase and linked to NE reassembly (Fernandez and Piano, 2006; Galy et al., 2006), and lamin B1 (a late nuclear reassembly marker) was not diminished (Figure S6, 13 and 18). Indeed, neither ELYS/MEL-28 nor lamin B1 was targeted to the chromatin of early mitotic cells expressing Repo-Man phospho-site mutants that exhibit premature targeting to the chromatin (Figure 4C, 1–12).

These abnormal distributions of Importin β, HP1α, and the formation of an abnormal Lamina appear to have their origin in an aberrant mitotic exit, rather than reflecting an interphase function of Repo-Man. Live cell imaging of a cell line stably expressing both GFP:Lamin A and H2B:mRFP showed that the abnormal lamina morphology appeared as cells exit mitosis. Moreover, cells depleted of Repo-Man never gained a smooth Lamina rim even when observed for up to 10 hr after cell division (Figure S5C, 1–3).

Results of RNAi rescue experiments are consistent with the hypothesis that the N terminus of Repo-Man is a module that functions in nuclear reassembly, whereas C-terminal Repo-Man functions in chromatin remodelling. Expression of GFP:Repo-Man^1–135^ in the RNAi background partially rescued both the nuclear shape defects and the abnormal distribution of Importin β in anaphase and interphase (Figure 7C and Figure S7). However, GFP:Repo-Man^1–135^, which does not bind PP1, was unable to rescue the HP1 mislocalization seen following Repo-Man RNAi (data not shown).

Overall, our studies reveal that Repo-Man functions both as a PP1 regulatory subunit in chromatin remodelling at the end of mitosis and in a PP1-independent pathway affecting early events during reassembly of the G1 nucleus.

## Discussion

### CDK Phosphorylation Regulates Repo-Man/PP1 Complex Localization and Function

In interphase, Repo-Man-PP1 is closely associated with nucleosomal histone H2B and plays an important role in regulating the DNA damage response (Peng et al., 2010). Upon mitotic entry, CdK1-cyclin B (possibly with other kinases) phosphorylates Repo-Man, decreasing its affinity for chromatin.

Several aspects of Repo-Man function are regulated by phosphorylation (Figure 7D). Phosphorylation at T412 prevents PP1 from binding to the RVTF motif. Based on similar observations with other mitotic phosphatase complexes including KNL1-PP1 (Liu et al., 2010; Welburn et al., 2010) and CENP-E-PP1 (Kim et al., 2010), we propose that phosphorylation at or near the PP1-binding motif constitutes a general mechanism for regulation of mitotic PP1 holoenzymes by mitotic kinases, including CDKs (for Repo-Man) and Aurora B (for KNL-1 and CENP-E).

Repo-Man localization is also regulated by phosphorylation. Repo-Man T412A can bind to chromosomes during early mitosis. However, if the T412A mutation is combined with a PP1-nonbinding RAXA mutation, then Repo-Man remains diffuse. This strongly suggests that Repo-Man:PP1 undergoes a two-step phosphoregulation during mitotic exit. At anaphase onset, T412 dephosphorylation by an as-yet-unidentified phosphatase allows PP1 binding. Next, PP1, or another phosphatase activated by the drop in CDK activity, dephosphorylates other sites on Repo-Man, converting it to a form that can bind anaphase chromatin.

This regulation of the Repo-Man:PP1 complex acts as a “fail safe device” as suggested previously for MYPT1:PP1 (Walker et al., 2000). Mitotic phosphorylation ensures that Repo-Man is released from the chromatin and also blocks the formation and activation of the phosphatase complex.

### Repo-Man Has Dual Functions in Anaphase

Repo-Man has two separate regions that direct it to distinct locations during mitotic exit. The C terminus targets the protein to anaphase chromatin, while the N terminus directs it to distinct foci at the chromosome periphery. These different localization patterns are temporally resolved during mitotic exit.

How can a single protein specifically target to two mutually exclusive locations? It may be that the C-terminal “general chromatin-binding” motif in Repo-Man has a higher affinity for chromosomes and that its chromosomal-binding sites must be saturated before the second motif can target the protein to the chromosome periphery. Alternatively, recognition of the second binding site might require another chromatin modification event, e.g., chromatin dephosphorylation by the early-binding population of Repo-Man:PP1. It is possible that Repo-Man binding to the chromosome periphery involves interactions with other as-yet-unidentified proteins.

Repo-Man docked on anaphase chromosomes contributes to the dephosphorylation of histone H3 at its mitotic phospho-sites (Thr3, Ser10, and Ser28). Dephosphorylation of H3Thr3 removes the chromatin docking site for the CPC (Kelly et al., 2010; Wang et al., 2010; Yamagishi et al., 2010). Thus, Repo-Man:PP1 activation at anaphase onset could promote CPC transfer from the chromatin to the central spindle and cleavage furrow (Ruchaud et al., 2007). This might be analogous to Cdc14 function in budding yeast, although Cdc14 acts on INCENP (Sli15) rather than H3 (Pereira and Schiebel, 2003). Premature targeting of mutant Repo-Man^TA3^:PP1 to the chromatin causes dephosphorylation of H3T3ph, resulting in CPC delocalization and chromosome alignment defects. Consistent with this, a recent study has shown that Repo-Man:PP1 can dephosphorylate H3T3 and cause delocalization of the CPC in early mitosis (Qian et al., 2011).

Depletion of Repo-Man interferes with H3Ser10 dephosphorylation in HeLa cells. H3S10ph has been reported to interfere with HP1 binding to H3K9me3 (Fischle et al., 2005; Hirota et al., 2005). Indeed, HP1α accumulation in foci on chromosomes is impaired during mitotic exit in Repo-Man-depleted cells. Thus, Repo-Man:PP1 dephosphorylation of H3Ser10 appears to be required for normal heterochromatin formation in G1 (Figure 7D).

The population of Repo-Man that accumulates at the chromosome periphery is important for normal NE reformation. The abnormal lobulated nuclear morphology observed following Repo-Man depletion by RNAi is a consequence of abnormal mitotic exit that occurs when Importin β localization is no longer correctly regulated by Repo-Man. We have shown that Repo-Man binds directly to Importin β and recruits it to sites at the chromosome periphery (Figure 7D). Importin β is an early factor involved in NE reassembly (Zhang et al., 2002; Clarke and Zhang, 2008), possibly also regulating NPC formation (Rotem et al., 2009). Rotem et al. (2009) predicted that additional regulators beside Importin β and Ran may be involved in coordinating the initial seeding of chromatin during NE reassembly. Repo-Man may be one such factor.

Importin β has recently been shown to associate with PP2A/B55α and function during mitotic exit either by targeting the phosphatase or acting as a molecular chaperone (Schmitz et al., 2010). However, the mechanism of its recruitment and positioning during nuclear reassembly was not previously understood. We show here that Repo-Man binding to Importin β is direct, resides within the N-terminal domain of Repo-Man (aa 1–135)—i.e., is independent of the PP1-binding activity of Repo-Man—and is inhibited by CDK-cyclin B phosphorylation. Repo-Man bound to the chromosome periphery could serve as an anchoring site for Importin β, potentially marking sites for NPC reassembly (Figure 7D).

Localized catalytic activity of Repo-Man:PP1 may also be required for later steps during NPC reassembly. Dephosphorylation of nucleoporins is essential in NPC reassembly (Onischenko et al., 2005), and we have identified NUP153 as a Repo-Man interactor. Moreover, recent work has also identified NUP153 as a PP1 substrate using an affinity chromatography approach in a search for PP1 nuclear substrates (Moorhead et al., 2008).

Importantly, Repo-Man binding to the chromosome periphery is not sufficient to trigger the complete pathway of NE reassembly. Other key proteins, including ELYS-MEL28 and lamins, are not recruited to chromatin by Repo-Man. We postulate that Repo-Man targeting of Importin β acts either downstream or in parallel to the ELYS-MEL28 pathway.

### Conclusions

The PP1γ-regulatory/targeting subunit Repo-Man is a modular protein that coordinates chromatin remodeling with nuclear envelope reassembly during mitotic exit (Figure 7D). A C-terminal Repo-Man module remodels the chromatin by targeting PP1 to anaphase chromosomes and directing the dephosphorylation of histone H3. This mobilizes the CPC and prepares the chromatin for heterochromatin assembly during G1. An N-terminal Repo-Man module binds Importin β and targets it to the periphery of the anaphase chromosomes. This is required for normal nuclear envelope assembly.

## Experimental Procedures

### Cell Culture, Cloning, and Transfections

hRepo-Man^WT^ and hRepo-Man^RAXA^ were cloned into the pTrAP vector (Samejima et al., 2008); DT40 cell lines were obtained by G418 selection (2 mg/ml). A HeLa cell line expressing H2B:mRFP and Lamin A:GFP was obtained by cotransfection of both constructs and selection in G418 (2 mg/ml). Cells positive for both GFP and RFP were selected by FACS.

Repo-Man mutants were generated by QuikChange Site-Directed Mutagenesis Kit (Agilent Technologies) using the plasmid GFP:Repo-Man (Table S1). The following primer sequences were used: T412A, 5′-tctttgccagcaaatgatccattgcgtaaagga-3′; T34A, 5′-actgggaagattgtggatcctcagaagcatgcc-3′; and T419A, 5′-ttgcgtaaaggaggagcacctgtttgtaaaaaa-3′. Repo-Man deletion mutants were generated by PCR, sequenced, and cloned into pEGFP-N1.

For the GFP:Laci-Repo-Man^WT^ and GFP:Laci-Repo-Man^1–135^ constructs, the Laci sequence was obtained by PCR and cloned into GFP:Repo-Man by XhoI/KpnI. Chicken PP1γ and β were obtained by RT-PCR and cloned into pEGFPN1. For a list of all new constructs generated, see Table S1.

Transient transfections for DT40 were conducted as previously described (Vagnarelli et al., 2006), and transient transfection in HeLa cells was performed by FuGENE 6 following the manufacturer's directions.

For quantification of mitotic localization (Figure 1D), cells were fixed with paraformaldehyde 24 hr posttransfection. For each transfected prometaphase-metaphase cell (50 cells), it was recorded if the GFP signal was diffuse in the cytoplasm as in Figure 1B, 2, or was present on the chromosomes as in Figure 1B, 5, 8, and 11.

For quantification of lamina morphology, Importin β staining, and HP1 localization, the control RNAi staining was used as a reference (for Lamin A Figure 7A, 6; for Importin β Figure 7B, 6; for HP1 Figure 6C, 6). A total of 300 nuclei and three separate experiments were averaged to produce Figure 6D.

### Indirect Immunofluorescence Microscopy

Cells were fixed in 4% PFA and processed as previously described (Vagnarelli et al., 2006). The antibodies used and dilutions are listed in Table S2. Fluorescence-labeled secondary antibodies were applied at 1:200 (Jackson ImmunoResearch). 3D data sets were acquired using a cooled CCD camera (CH350; Photometrics) on a wide-field microscope (DeltaVision Spectris; Applied Precision) with a NA 1.4 Plan Apochromat lens. The data sets were deconvolved with softWoRx (Applied Precision). Three-dimensional data sets were converted to Quick Projection in softWoRx, exported as TIFF files, and imported into Adobe Photoshop for final presentation.

Live cell imaging was performed with a DeltaVision microscope as previously described (Vagnarelli et al., 2006).

For quantification of H3 phosphorylation levels on chromosomes, images of prometaphases from transfected and untransfected cells were acquired from the same slide, and the intensity of chromosomal staining for each anti-phospho-H3 antibody was determined. The 3-dimensional data sets obtained at the same exposure for transfected and untransfected cells were projected as mean intensity. A 10 × 10 pixel area contained within the chromosomes was used to measure the total intensity of the signal. Ten different measurements per mitosis on different chromosomes were collected and averaged. An area of the same size was used to identify the background signal in each metaphase, and this value was subtracted from the measurement of the chromosome area. The staining intensity of the transfected cells was normalized relative to the untransfected cells on each slide.

For quantification of the H3 phosphorylation levels following Repo-Man RNAi, cells were stained with α-tubulin and H3Ser10ph. Only cells joined by a midbody were analyzed. The 3D data were projected as mean intensity, and the total fluorescence signal of a square including the entire nucleus was analyzed. A square of the same size was used to calculate the background adjacent to the cell, and the values were subtracted from the nuclear measurement.

### Quantitative Immunoblotting

Membranes were incubated with primary antibodies recognizing α-tubulin, H3S10ph, H3T3ph, and subsequently with IRDye-labeled secondary antibodies (LI-COR). Fluorescence intensities were subsequently determined using an LI-COR Odyssey CCD scanner according to the manufacturer's instructions (LI-COR Biosciences).

### In vitro Phosphorylation

GST-Repo-Man^166–659^ or GST-Repo-Man^1–135^ was expressed in *E*. *coli* and purified on a glutathione-Sepharose column. After elution, the recombinant protein was incubated with 100 μM ^32^P-ATP and Cdk1-cyclin B in the manufacturer kinase buffer (NEB) at 30°C for 1 hr. The reaction was stopped by addition of SDS sample buffer, separated by SDS-PAGE, and stained with Coomassie blue. The gel was dried on 3MM paper and exposed to X-ray film.

### In vitro Binding Assay

His-tagged Importin β (gift of M. Platani, Edinburgh) was expressed in *E*. *coli* and purified on TALON beads. We incubated 15 μl of beads with GST-Repo-Man^1–135^ or GST alone for 1 hr at 4°C in Binding Buffer (20 mM HEPES [pH 7.4], 110 mM K Acetate, 2 mM Mg Acetate, 0.2 mM DTT). Beads were washed three times in Binding Buffer and boiled in SDS sample buffer before separation on SDS-PAGE followed by Coomassie blue staining.

### RNAi

A 21-mer oligonucleotide (CGUACGCGGAAUACUUCGAdTdT) was used as a control (Elbashir et al., 2001). For Repo-Man RNAi, 5-′UGACAGACUUGACCAGAAATT-3′ with a 5′Cy5 labeled was used. A second oligonucleotide, 5′-CCUAAUAAUCAUCAAUCU-3′, was also used to confirm the phenotypes.

HeLa cells in exponential growth were seeded onto polylysine-coated glass coverslips and grown overnight in RPMI/10% FBS. RNAi was performed as previously described (Elbashir et al., 2001).

For the rescue experiment, RNAi was performed using Polyplus jetPRIME (PEQLAB). HeLa cells at 50% confluence were transfected with 400 ng of plasmid DNA and 50 nM of SiRNA oligonucleotides. Analyses were carried out 48 hr posttransfection.

### Pull-Down Experiments and Mass Spectrometry Analysis

Cell lines stably expressing TrAP:hRepo-Man^WT^, TrAP:hRepo-Man^RAXA^, and TrAP:GFP were incubated with colcemid overnight. After 18 hr, 50 mM roscovitine was added to the cultures for 10 min where indicated. A total of 1 × 10^8^ cells was collected by centrifugation and resuspended in Buffer A (75 mM Tris:HCl [pH 7.4], 40 mM KCl, 1 mM K-EDTA [pH 7.4], 0.3 mM Spermidine, 0.2 mM Spermine plus protease and phosphatase inhibitors) for 5 min at room temperature. The pellet was then resuspended in 2× Buffer A plus digitonin and lysed on ice by Dounce homogenization. The chromosomes were collected on a sucrose cushion, washed in 1× Buffer A, and resuspended in lysis buffer (50 mM Tris:HCl [pH 7.4], 200 mM NaCl, 0.5% NP40, and protease and phosphatase inhibitors). The samples were sonicated, and the soluble fraction was collected after centrifugation. The lysate was incubated with streptavidin beads for 1 hr at 4°C, washed three times, and eluted with D-Biotin for 20 min. The elutes were bound to a S-column for 1 hr. After washes, the bound fraction was eluted with SDS sample buffer, separated by a short SDS-PAGE run, and subjected to mass spectrometry analysis.

For SILAC analyses, the cell lines were grown in RPMI(-)Arg(-)Lys supplemented with 100 ng/ml ^13^C Lysine plus 30 nm/ml ^13^C Arginine (Cambridge Isotope Laboratories) for the heavy labeling, or ^12^C Lysine/^12^C Arginine for the light labeling in media supplemented with 10% dialyzed FBS.

### Mass Spectrometry Analysis

For all mass spectrometric analyses, in-gel digestion was performed as described (Shevchenko et al., 2006). Digested material was cleaned up on a reverse-phase C18 StageTip (Rappsilber et al., 2003) and reduced to a 5 μl volume by vacuum evaporation. All samples were analyzed on an LTQ-Orbitrap Classic (Thermo Fisher), connected to an HTC-PAL auto-sampler (CTC Analytics), and a 1200 nanoHPLC pump (Agilent). Gradients (5%–20% acetonitrile in 90 min) ran at 300 nl/min over a C18-packed pico-spray emitter (Proxeon).

Mass spectrometric data were acquired in cycles of one FT-full scan (30,000 resolution) in the Orbitrap and up to six MS2-events in the LTQ-ion trap. Identification and quantification were performed using MaxQuant v 1.0.11.1 for processing and Mascot for searches. Peak lists of individual runs were generated using MaxQuant with the variable modifications oxidation (M), acetylation (K), and fixed modification carbamidomethylation (C), and the doublet quantification mode for arg-6 and lys-6. Precursor mass tolerance was set to 7 ppm (default). For phosphorylation searches, DTASuperCharge v 1.19 was used at default parameters to generate peak lists. The peak data were searched using Mascot as above except using Phospho (STY) as additional variable modification and mass tolerance set to 10 ppm. Peak lists were searched against IPI-chicken (quantitative pull down) or a custom database containing the sequence of GST-tagged H2B (for phosphorylation identification) using Mascot v 2.1.

### FLIM/FRET

FLIM experiments were performed on a Leica SP5 laser-scanning confocal microscope equipped with a Spectra-Physics Mai-Tai multiphoton laser (700–1020 nm). GFP:Repo-Man was imaged using multiphoton excitation wavelength of 890 nm. Emission detection was through a 500–550 nm band-pass filter (Chroma). Fluorescence lifetimes were measured using a Becker & Hickel PMC-100 external detector and SPC/830 acquisition card controlled by SPCM software for time-correlated single photon counting. Images were analyzed using SPCImage (Becker & Hickel).

Lifetimes of GFP alone and GFP:Repo-Man ± H2B:mRFP were measured.

Data were collected from a minimum of 20 cells for each experimental condition.

## Supplemental Information

Document S1. Seven Figures and Two Tables

Movie S1. GFP:Repo-Man ^403–1023^ Localizes to Bulk Chromatin after Anaphase Onset Dt40 cells transiently transfected with GFP:Repo-Man^403–1023^ and H2B:mRFP were imaged every minute from prometaphase to telophase-cytokinesis.

Movie S2. GFP:Repo-Man^1–397^ Localizes to the Chromosomes Periphery in Telophase Dt40 cells transiently transfected with GFP:Repo-Man^403–1023^ and H2B:mRFP were imaged every minute from prometaphase to telophase-cytokinesis.

## References

[bib1] Adhvaryu K.K., Selker E.U. (2008). Protein phosphatase PP1 is required for normal DNA methylation in *Neurospora*. Genes Dev..

[bib2] Axton J.M., Dombrádi V., Cohen P.T., Glover D.M. (1990). One of the protein phosphatase 1 isoenzymes in *Drosophila* is essential for mitosis. Cell.

[bib3] Bannister A.J., Zegerman P., Partridge J.F., Miska E.A., Thomas J.O., Allshire R.C., Kouzarides T. (2001). Selective recognition of methylated lysine 9 on histone H3 by the HP1 chromo domain. Nature.

[bib4] Bayliss R., Sardon T., Vernos I., Conti E. (2003). Structural basis of Aurora-A activation by TPX2 at the mitotic spindle. Mol. Cell.

[bib5] Cantin G.T., Yi W., Lu B., Park S.K., Xu T., Lee J.D., Yates J.R. (2008). Combining protein-based IMAC, peptide-based IMAC, and MudPIT for efficient phosphoproteomic analysis. J. Proteome Res..

[bib6] Chen F., Archambault V., Kar A., Lio’ P., D'Avino P.P., Sinka R., Lilley K., Laue E.D., Deak P., Capalbo L., Glover D.M. (2007). Multiple protein phosphatases are required for mitosis in *Drosophila*. Curr. Biol..

[bib7] Clarke P.R., Zhang C. (2008). Spatial and temporal coordination of mitosis by Ran GTPase. Nat. Rev. Mol. Cell Biol..

[bib8] Dephoure N., Zhou C., Villén J., Beausoleil S.A., Bakalarski C.E., Elledge S.J., Gygi S.P. (2008). A quantitative atlas of mitotic phosphorylation. Proc. Natl. Acad. Sci. USA.

[bib9] De Wulf P., Montani F., Visintin R. (2009). Protein phosphatases take the mitotic stage. Curr. Opin. Cell Biol..

[bib10] Dultz E., Zanin E., Wurzenberger C., Braun M., Rabut G., Sironi L., Ellenberg J. (2008). Systematic kinetic analysis of mitotic dis- and reassembly of the nuclear pore in living cells. J. Cell Biol..

[bib11] Elbashir S.M., Lendeckel W., Tuschl T. (2001). RNA interference is mediated by 21- and 22-nucleotide RNAs. Genes Dev..

[bib12] Eyers P.A., Erikson E., Chen L.G., Maller J.L. (2003). A novel mechanism for activation of the protein kinase Aurora A. Curr. Biol..

[bib13] Fernandez A.G., Piano F. (2006). MEL-28 is downstream of the Ran cycle and is required for nuclear-envelope function and chromatin maintenance. Curr. Biol..

[bib14] Fischle W., Tseng B.S., Dormann H.L., Ueberheide B.M., Garcia B.A., Shabanowitz J., Hunt D.F., Funabiki H., Allis C.D. (2005). Regulation of HP1-chromatin binding by histone H3 methylation and phosphorylation. Nature.

[bib15] Galy V., Askjaer P., Franz C., López-Iglesias C., Mattaj I.W. (2006). MEL-28, a novel nuclear-envelope and kinetochore protein essential for zygotic nuclear-envelope assembly in C. elegans. Curr. Biol..

[bib16] Güttinger S., Laurell E., Kutay U. (2009). Orchestrating nuclear envelope disassembly and reassembly during mitosis. Nat. Rev. Mol. Cell Biol..

[bib17] Hirota T., Kunitoku N., Sasayama T., Marumoto T., Zhang D., Nitta M., Hatakeyama K., Saya H. (2003). Aurora-A and an interacting activator, the LIM protein Ajuba, are required for mitotic commitment in human cells. Cell.

[bib18] Hirota T., Lipp J.J., Toh B.H., Peters J.M. (2005). Histone H3 serine 10 phosphorylation by Aurora B causes HP1 dissociation from heterochromatin. Nature.

[bib19] Hsu J.Y., Sun Z.W., Li X., Reuben M., Tatchell K., Bishop D.K., Grushcow J.M., Brame C.J., Caldwell J.A., Hunt D.F. (2000). Mitotic phosphorylation of histone H3 is governed by Ipl1/aurora kinase and Glc7/PP1 phosphatase in budding yeast and nematodes. Cell.

[bib20] Ito H., Koyama Y., Takano M., Ishii K., Maeno M., Furukawa K., Horigome T. (2007). Nuclear envelope precursor vesicle targeting to chromatin is stimulated by protein phosphatase 1 in *Xenopus* egg extracts. Exp. Cell Res..

[bib21] Kelly A.E., Ghenoiu C., Xue J.Z., Zierhut C., Kimura H., Funabiki H. (2010). Survivin reads phosphorylated histone H3 threonine 3 to activate the mitotic kinase Aurora B. Science.

[bib22] Kim Y., Holland A.J., Lan W., Cleveland D.W. (2010). Aurora kinases and protein phosphatase 1 mediate chromosome congression through regulation of CENP-E. Cell.

[bib23] Lachner M., O'Carroll D., Rea S., Mechtler K., Jenuwein T. (2001). Methylation of histone H3 lysine 9 creates a binding site for HP1 proteins. Nature.

[bib24] Liu D., Vleugel M., Backer C.B., Hori T., Fukagawa T., Cheeseman I.M., Lampson M.A. (2010). Regulated targeting of protein phosphatase 1 to the outer kinetochore by KNL1 opposes Aurora B kinase. J. Cell Biol..

[bib25] Malik R., Lenobel R., Santamaria A., Ries A., Nigg E.A., Körner R. (2009). Quantitative analysis of the human spindle phosphoproteome at distinct mitotic stages. J. Proteome Res..

[bib26] Mayya V., Lundgren D.H., Hwang S.I., Rezaul K., Wu L., Eng J.K., Rodionov V., Han D.K. (2009). Quantitative phosphoproteomic analysis of T cell receptor signaling reveals system-wide modulation of protein-protein interactions. Sci. Signal..

[bib27] Moorhead G.B., Trinkle-Mulcahy L., Nimick M., De Wever V., Campbell D.G., Gourlay R., Lam Y.W., Lamond A.I. (2008). Displacement affinity chromatography of protein phosphatase one (PP1) complexes. BMC Biochem..

[bib28] Olsen J.V., Vermeulen M., Santamaria A., Kumar C., Miller M.L., Jensen L.J., Gnad F., Cox J., Jensen T.S., Nigg E.A. (2010). Quantitative phosphoproteomics reveals widespread full phosphorylation site occupancy during mitosis. Sci. Signal..

[bib29] Onischenko E.A., Gubanova N.V., Kiseleva E.V., Hallberg E. (2005). Cdk1 and okadaic acid-sensitive phosphatases control assembly of nuclear pore complexes in *Drosophila* embryos. Mol. Biol. Cell.

[bib30] Peng A., Lewellyn A.L., Schiemann W.P., Maller J.L. (2010). Repo-man controls a protein phosphatase 1-dependent threshold for DNA damage checkpoint activation. Curr. Biol..

[bib31] Pereira G., Schiebel E. (2003). Separase regulates INCENP-Aurora B anaphase spindle function through Cdc14. Science.

[bib32] Qian J., Lesage B., Beullens M., Van Eynde A., Bollen M. (2011). PP1/Repo-Man Dephosphorylates Mitotic Histone H3 at T3 and Regulates Chromosomal Aurora B Targeting. Curr. Biol..

[bib33] Queralt E., Uhlmann F. (2008). Cdk-counteracting phosphatases unlock mitotic exit. Curr. Opin. Cell Biol..

[bib34] Rappsilber J., Ishihama Y., Mann M. (2003). Stop and go extraction tips for matrix-assisted laser desorption/ionization, nanoelectrospray, and LC/MS sample pretreatment in proteomics. Anal. Chem..

[bib35] Rotem A., Gruber R., Shorer H., Shaulov L., Klein E., Harel A. (2009). Importin beta regulates the seeding of chromatin with initiation sites for nuclear pore assembly. Mol. Biol. Cell.

[bib36] Ruchaud S., Carmena M., Earnshaw W.C. (2007). Chromosomal passengers: conducting cell division. Nat. Rev. Mol. Cell Biol..

[bib37] Samejima K., Ogawa H., Cooke C.A., Hudson D.F., Macisaac F., Ribeiro S.A., Vagnarelli P., Cardinale S., Kerr A., Lai F. (2008). A promoter-hijack strategy for conditional shutdown of multiply spliced essential cell cycle genes. Proc. Natl. Acad. Sci. USA.

[bib38] Schmitz M.H., Held M., Janssens V., Hutchins J.R., Hudecz O., Ivanova E., Goris J., Trinkle-Mulcahy L., Lamond A.I., Poser I. (2010). Live-cell imaging RNAi screen identifies PP2A-B55alpha and importin-beta1 as key mitotic exit regulators in human cells. Nat. Cell Biol..

[bib39] Shevchenko A., Tomas H., Havlis J., Olsen J.V., Mann M. (2006). In-gel digestion for mass spectrometric characterization of proteins and proteomes. Nat. Protoc..

[bib40] Steen R.L., Martins S.B., Taskén K., Collas P. (2000). Recruitment of protein phosphatase 1 to the nuclear envelope by A-kinase anchoring protein AKAP149 is a prerequisite for nuclear lamina assembly. J. Cell Biol..

[bib41] Terrak M., Kerff F., Langsetmo K., Tao T., Dominguez R. (2004). Structural basis of protein phosphatase 1 regulation. Nature.

[bib42] Trinkle-Mulcahy L., Andersen J., Lam Y.W., Moorhead G., Mann M., Lamond A.I. (2006). Repo-Man recruits PP1 gamma to chromatin and is essential for cell viability. J. Cell Biol..

[bib43] Vagnarelli P., Hudson D.F., Ribeiro S.A., Trinkle-Mulcahy L., Spence J.M., Lai F., Farr C.J., Lamond A.I., Earnshaw W.C. (2006). Condensin and Repo-Man-PP1 co-operate in the regulation of chromosome architecture during mitosis. Nat. Cell Biol..

[bib44] Walker K.S., Watt P.W., Cohen P. (2000). Phosphorylation of the skeletal muscle glycogen-targetting subunit of protein phosphatase 1 in response to adrenaline in vivo. FEBS Lett..

[bib45] Wang F., Dai J., Daum J.R., Niedzialkowska E., Banerjee B., Stukenberg P.T., Gorbsky G.J., Higgins J.M. (2010). Histone H3 Thr-3 phosphorylation by Haspin positions Aurora B at centromeres in mitosis. Science.

[bib46] Welburn J.P., Vleugel M., Liu D., Yates J.R., Lampson M.A., Fukagawa T., Cheeseman I.M. (2010). Aurora B phosphorylates spatially distinct targets to differentially regulate the kinetochore-microtubule interface. Mol. Cell.

[bib47] Yamagishi Y., Honda T., Tanno Y., Watanabe Y. (2010). Two histone marks establish the inner centromere and chromosome bi-orientation. Science.

[bib48] Zhang C., Hutchins J.R., Mühlhäusser P., Kutay U., Clarke P.R. (2002). Role of importin-beta in the control of nuclear envelope assembly by Ran. Curr. Biol..

