# Supplementary material for: Repo-Man Coordinates Chromosomal Reorganization with Nuclear Envelope Reassembly during Mitotic Exit
Source: Dev Cell. 2011 Aug 16;21-248(2):328–42. doi: 10.1016/j.devcel.2011.06.020 (PMC3480639; doi:10.1016/j.devcel.2011.06.020)
Supplement: Document S1. Seven Figures and Two Tables [file mmc1.pdf]

Developmental Cell, Volume *21*

Supplemental Information

Repo-Man Coordinates Chromosomal Reorganization with Nuclear Envelope Reassembly during Mitotic Exit

Paola Vagnarelli, Susana Ribeiro, Lau Sennels, Luis Sanchez-Pulido, Flavia de Lima Alves, Toon Verheyen, David A. Kelly, Chris P. Ponting, Juri Rappsilber, and William C. Earnshaw

A

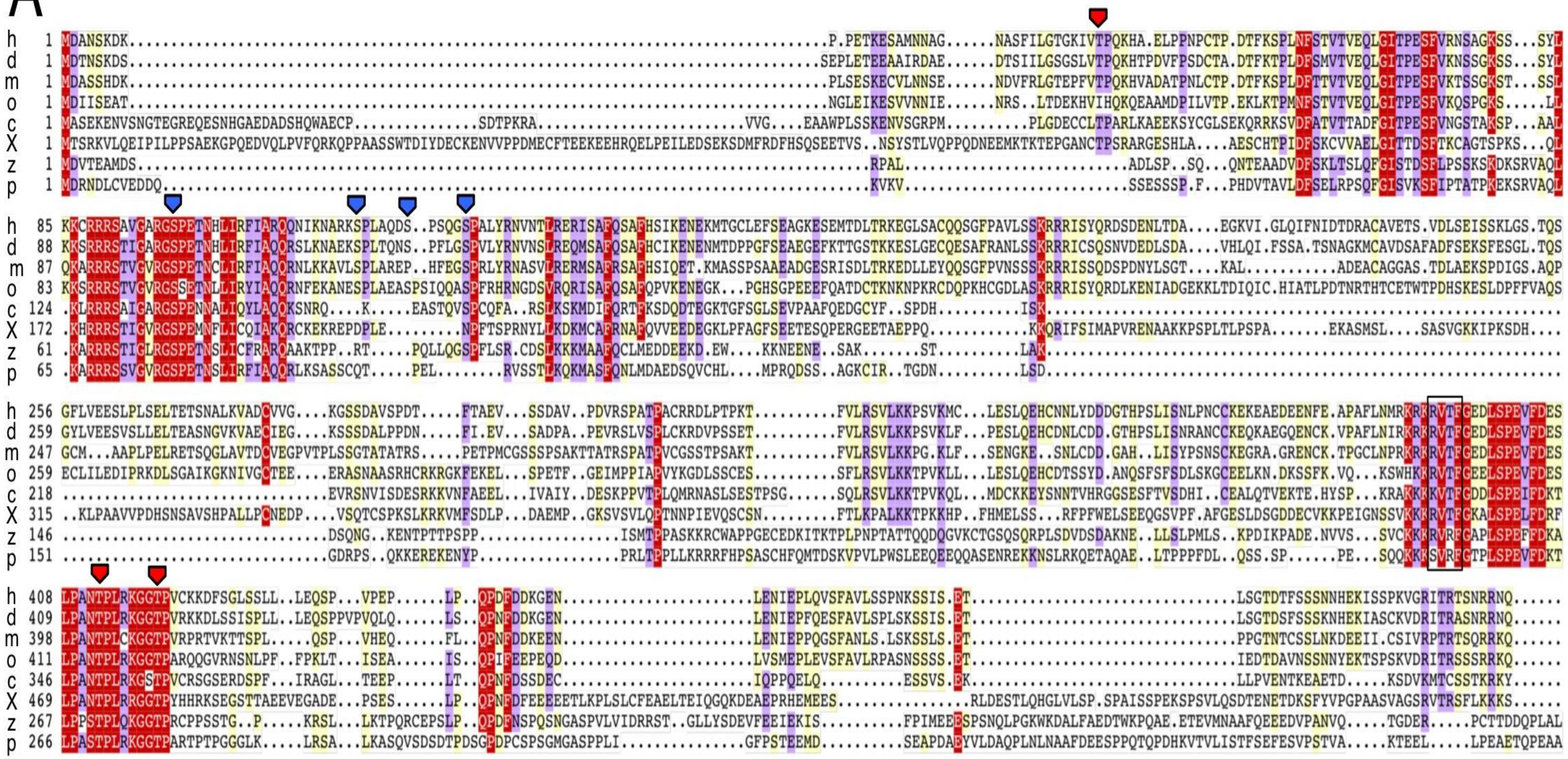

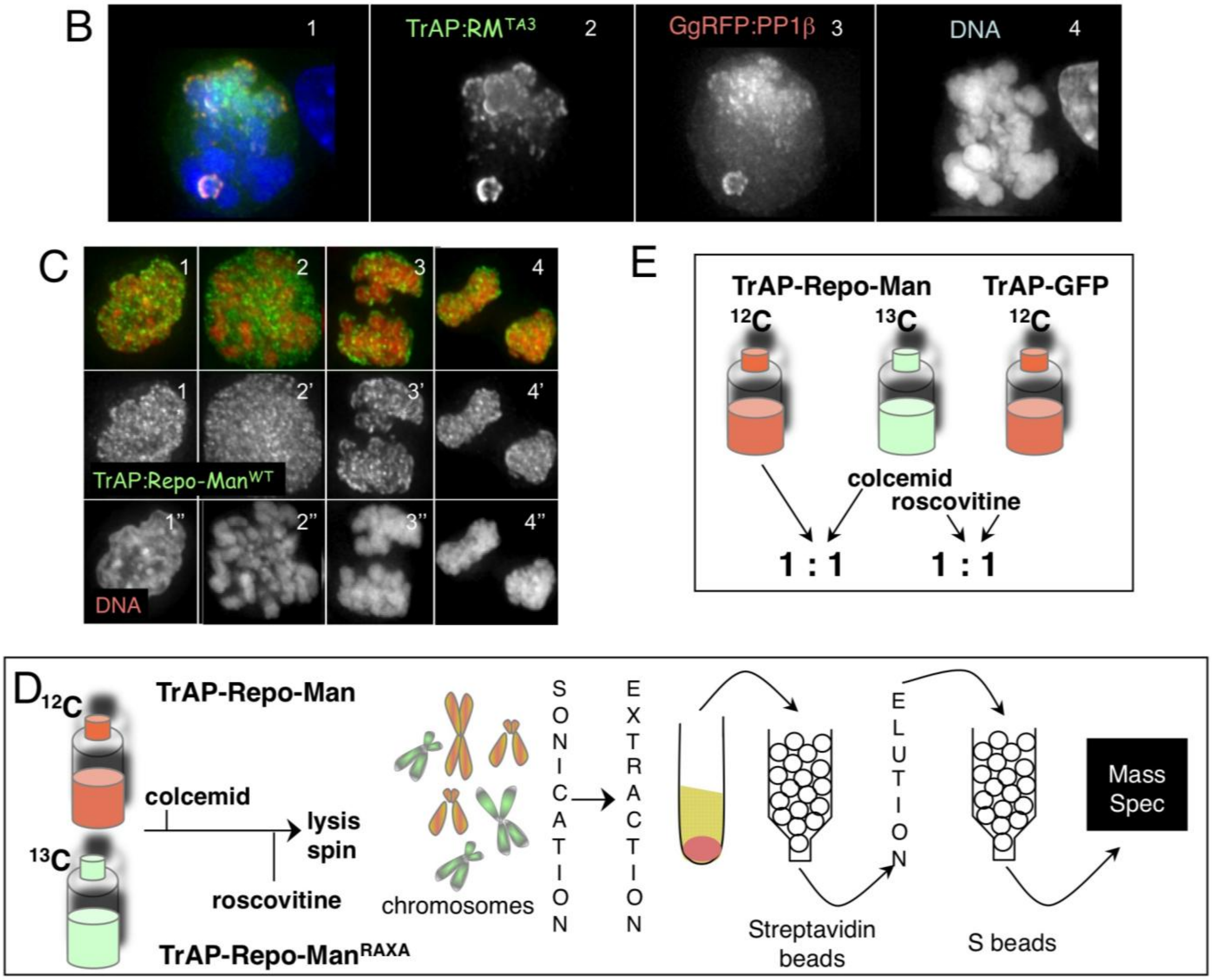

Figure 1. Repo-Man Phosphorylation Abolishes the Binding with PP1

(A) Multiple sequence alignment of Repo-Man from representative vertebrate species. The amino acid coloring scheme indicates average BLOSUM62 scores (which are correlated with amino acid conservation) for each alignment column: Red (greater than 3),violet (between 3 and 1.5) and yellow (between 1.5 and 0.5).

(h) *Homo sapiens*; (d) *Canis lupus familiaris*; (m) *Mus musculus*; (o) *Monodelphis domestica*; (c) *Gallus gallus*; (x) *Xenopus laevis*; (z) *Danio rerio*; (p) *Tetraodon nigroviridis*. The black box indicates the RTVF motif, red arrows indicate CDK sites mutated to Ala, blue arrows indicate known mitotic phosphorylations within the N-terminal region of Repo-Man.

(B) RFP-GgPP1β (red) colocalizes with GFP:Repo-Man^{TA3} (green) in transfected DT40 cells. (C) TrAP:hRepo-Man (stained with an anti S antibody -green) localizes normally in DT40 cells.

(D) Experimental design for pull-down experiments using cell lines expressing TrAP:hRepo-Man^{wt} and TrAP:hRepo-Man^{RAXA}.

(E) Experimental design for SILAC experiments to compare mitotic versus “anaphase” interactors.

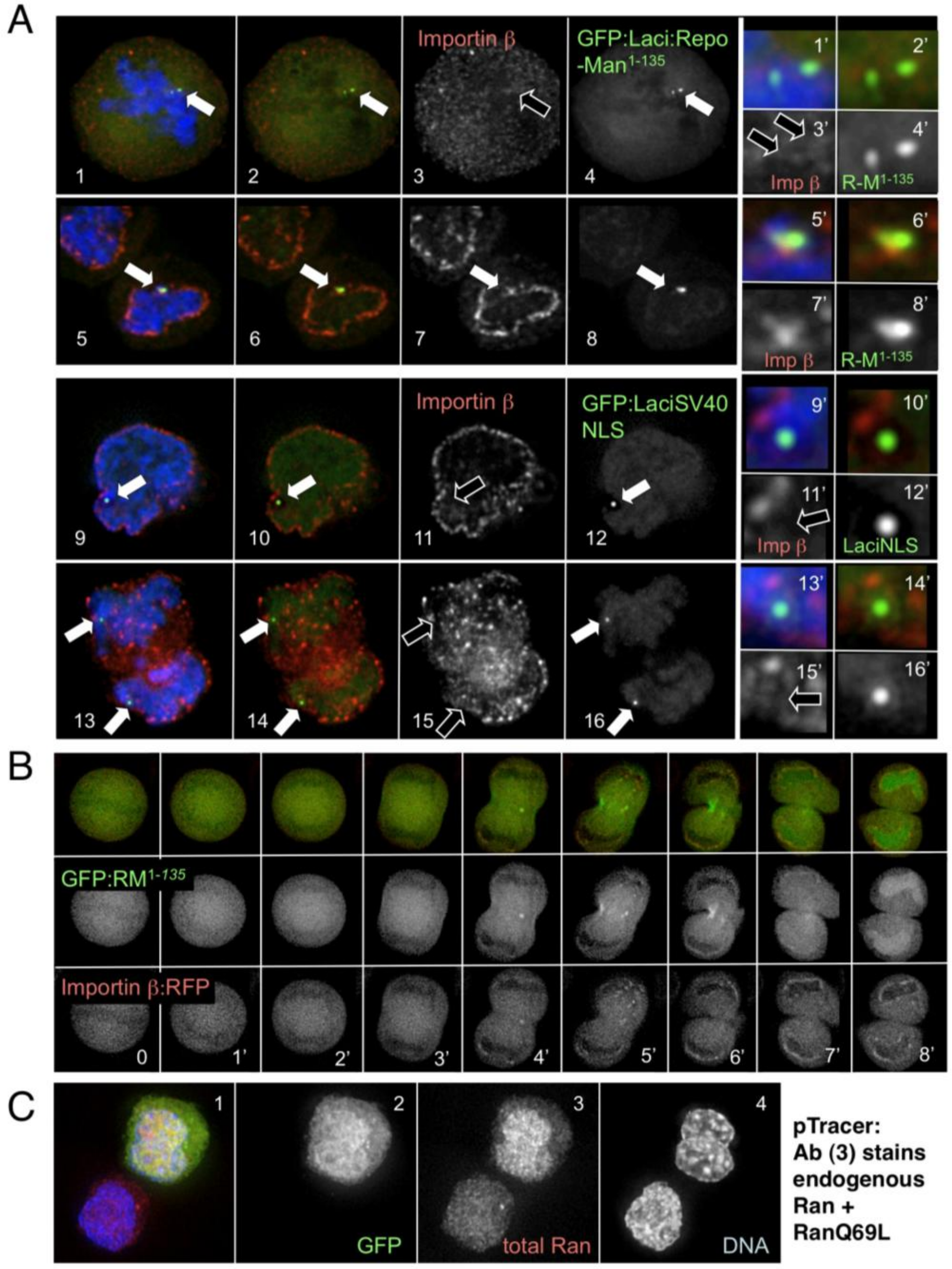

Figure 2. Repo-Man$^{1-135}$ Colocalizes with Importin β from Anaphase
(A) GFP:Laci-Repo-Man$^{1-135}$ (green) was expressed in DT40 cells carrying a LacO integration on a single chromosome (1-8). Importin β (red) does not co-localize with Repo-Man$^{1-135}$ in prometaphase-metaphase (1-4) but does in telophase (5-8). Panels (9-16), DT40 cells carrying a LacO integration on a single chromosome and expressing GFP:Laci-SV40NLS were stained for Importin β (red). Black arrows indicate the absence of Importin β targeting during early mitosis by GFP:Laci:Repo-Man$^{1-135}$ (3') or by the Laci-SV40NLS construct (11', 15').
(B) Both GFP:Repo-Man$^{1-135}$ (green) and RFP-Importin β (red) are recruited to the chromosome periphery at the same time and locations (4') after anaphase onset in transfected DT40 cells.
(C) Ran (antibody stain - red) is overexpressed in a cell transfected with pTracer:RANQ69L. GFP (green), DNA (blue).

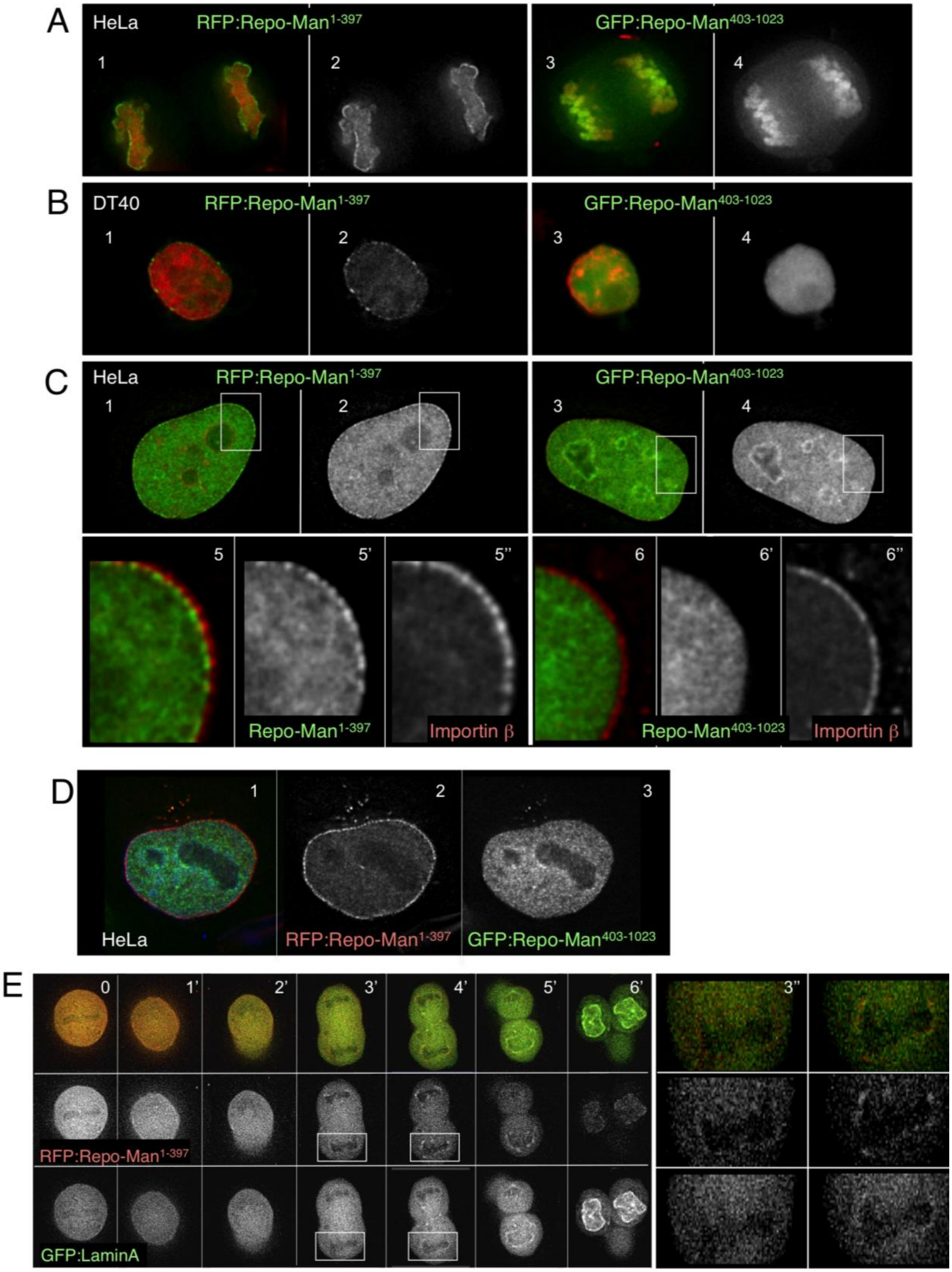
A
HeLa
RFP:Repo-Man1-397
GFP:Repo-Man403-1023
1
2
3
4
B
DT40
RFP:Repo-Man1-397
GFP:Repo-Man403-1023
1
2
3
4
C
HeLa
RFP:Repo-Man1-397
GFP:Repo-Man403-1023
1
2
3
4
5
5'
5''
6
6'
6''
Repo-Man1-397
Importin β
Repo-Man403-1023
Importin β
D
1
2
3
HeLa
RFP:Repo-Man1-397
GFP:Repo-Man403-1023
E
0
1'
2'
3'
4'
5'
6'
3''
RFP:Repo-Man1-397
GFP:LaminA

Figure 3. Distinct Repo-Man Chromatin Targeting Domains Localize Repo-Man to Different Chromosomal and Nuclear Regions

(A) Anaphase HeLa cells expressing GFP:Repo-Man$^{1\text{-}397}$ (1,2) and GFP:Repo-Man$^{403\text{-}1023}$ (3,4) (green).

(B) Nuclei of DT40 cells expressing GFP:Repo-Man$^{1\text{-}397}$ (1,2) and GFP:Repo-Man$^{403\text{-}1023}$ (3,4) (green).

(C) Nuclei of HeLa cells expressing GFP:Repo-Man$^{1\text{-}397}$ (1,2) or GFP:Repo-Man$^{403\text{-}1023}$ (3,4) (green) and stained for Importin β (red). Note the enrichment at the nuclear periphery for GFP:Repo-Man$^{1\text{-}397}$ (all panels – 1,2). Panels (5-5") and (6-6") show enlargements of the nuclear periphery from the boxed regions in (1-4).

(D) Nucleus of DT40 cell co-expressing RFP-Repo-Man$^{1\text{-}397}$ (red) and GFP:Repo-Man$^{403\text{-}1023}$ (green). The two fragments are enriched in different nuclear compartments.

(E) Stills from a movie showing the accumulation of RFP-Repo-Man$^{1\text{-}397}$ (red) and GFP:Lamin A (green) at the chromosome periphery during mitotic exit. At 3' only RFP-Repo-Man$^{1\text{-}397}$ (red) is accumulating around the chromosomes while Lamin A starts appearing at 4' (insets in 3' and 4').

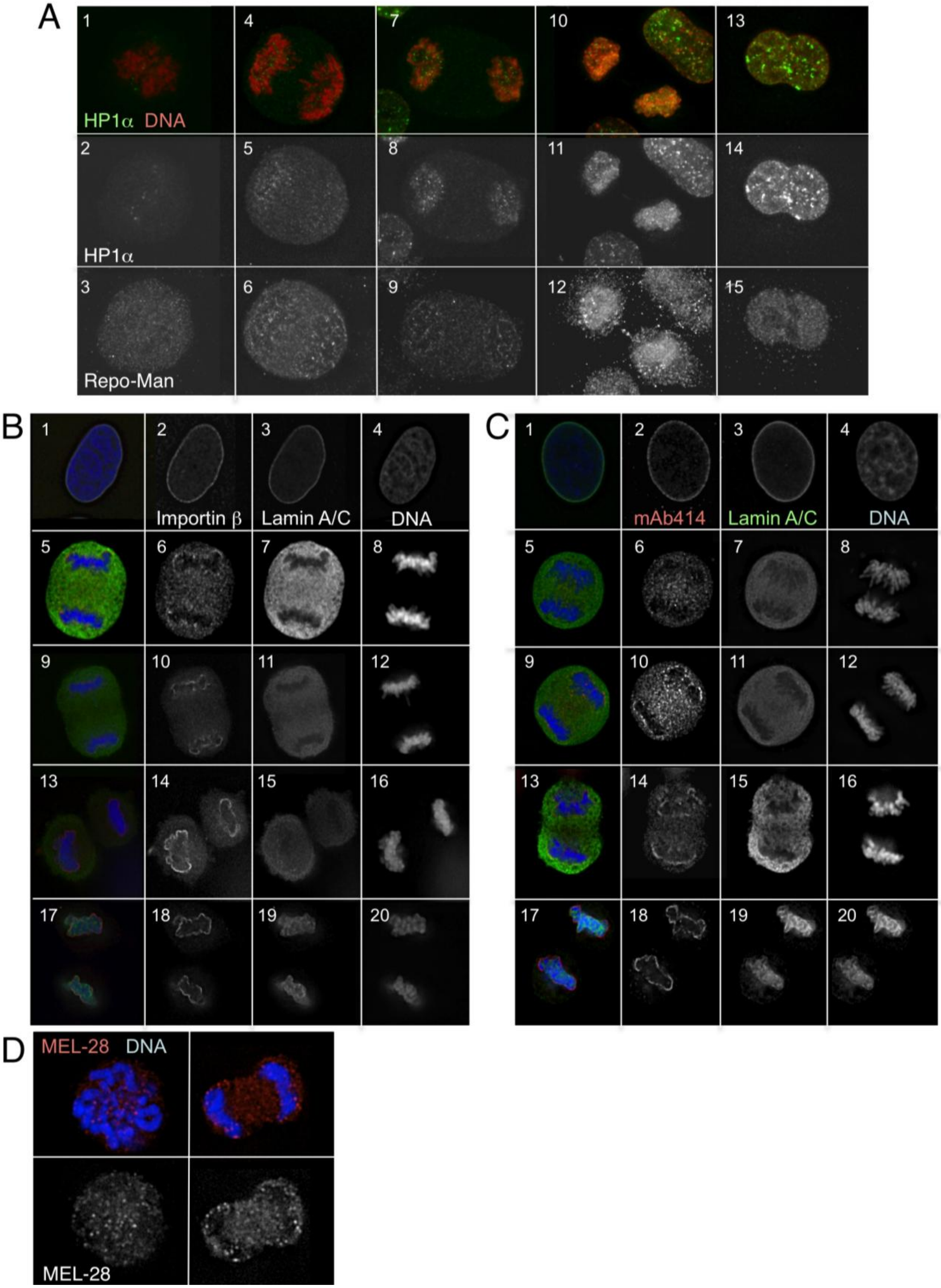
A
1
4
7
10
13
HP1α DNA
2
5
8
11
14
HP1α
3
6
9
12
15
Repo-Man
B
1
2
3
4
Importin β
Lamin A/C
DNA
5
6
7
8
9
10
11
12
13
14
15
16
17
18
19
20
C
1
2
3
4
mAb414
Lamin A/C
DNA
5
6
7
8
9
10
11
12
13
14
15
16
17
18
19
20
D
MEL-28 DNA
MEL-28

Figure 4. Recruitment of Importin β, mAb414 Antigens, Lamin A and HP1α onto the Chromosomes during Mitotic Exit

(A) HP1α is re-loaded on the chromatin during late anaphase-telophase.

(B) Importin β and (C) mAb414 antigens are recruited around the segregating chromosomes in anaphase and before the accumulation of Lamin A/C becomes clearly visible.

(D) MEL-28 staining (red) in prometaphase-metaphase and anaphase.

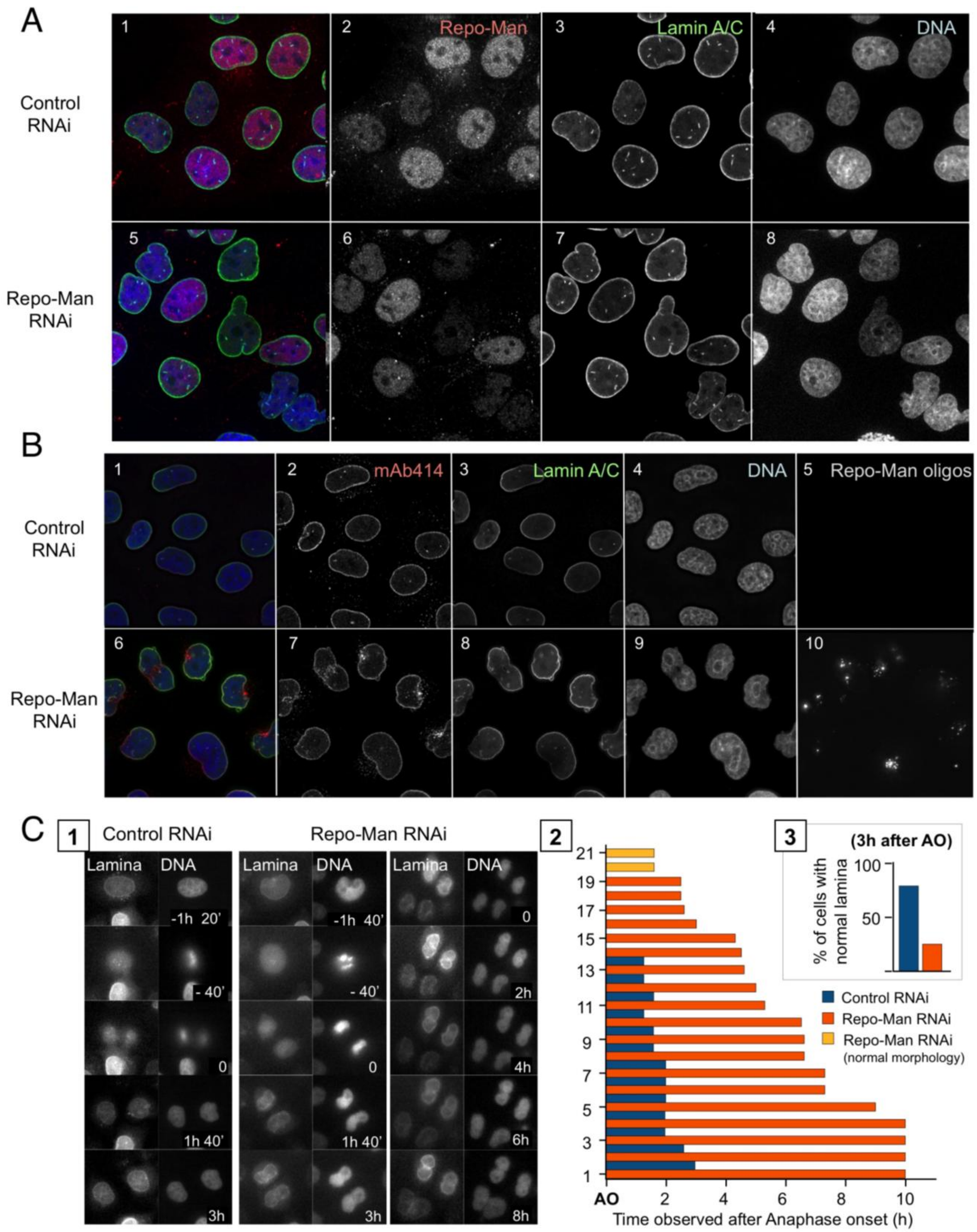
A
Control RNAi
Repo-Man RNAi
Repo-Man
Lamin A/C
DNA
B
Control RNAi
Repo-Man RNAi
mAb414
Lamin A/C
DNA
Repo-Man oligos
C
Control RNAi
Repo-Man RNAi
Lamina
DNA
-1h 20'
- 40'
0
1h 40'
3h
-1h 40'
2h
4h
6h
8h
Time observed after Anaphase onset (h)
AO
(3h after AO)
% of cells with normal lamina
Control RNAi
Repo-Man RNAi
Repo-Man RNAi (normal morphology)

Figure 5. Repo-Man RNAi Causes Abnormal Nuclear Morphology and Mislocalisation of mAb414 Antigens
(A) Repo-Man RNAi using a second oligonucleotide: CCUAAUAAUCAUCAAUCU (5-8) causes the appearance of cells with abnormal nuclear morphology, confirming results shown in the other figures using the published oligonucleotide (Trinkle-Mulcahy et al., 2006).
(B) Repo-Man RNAi in HeLa cells causes an abnormal distribution of mAb414 antigens (6-10).
(C) HeLa cells expressing Lamin A:GFP and H2B:mRFP were transfected with control or Repo-Man RNAi oligonucleotides. Starting 24h later, the cells were imaged every 20' for 22 h. Videos were analysed, recording the time from anaphase onset required for the re-formation of a smooth round envelope (judged by lamin A). (1) Examples of cell division in control and Repo-Man RNAi. (2) Quantitation of the results for 13 control and 21 Repo-Man RNAi cells. Two of the latter reformed normal envelopes. The other 19 cells after Repo-Man RNAi never reformed normal envelopes during the indicated times of imaging. (3) Percentage of cells with normal lamina at 3h post mitosis.

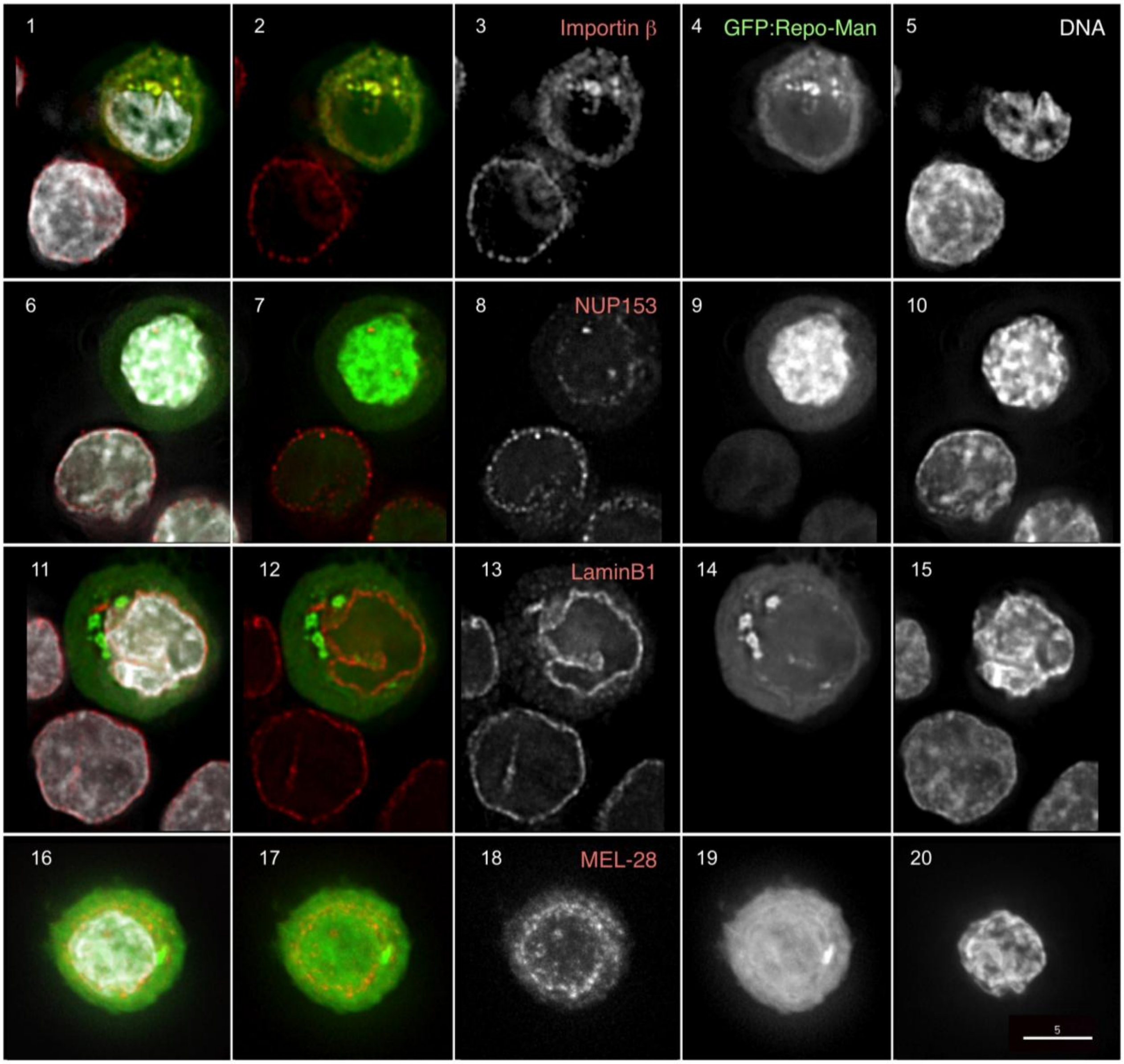

Figure 6. Repo-Man Overexpression Alters Importin β and Nup153 Distribution at the Nuclear Rim

DT40 cells over-expressing GFP:hRepo-Man (green) were stained for Importin β (1-5), Nup153 (6-10), LaminB1 (11-15) and MEL-28 (16-20) (red). Repo-Man highly over-expressing cells show a reduction in the nuclear rim staining for Importin β (1-5 top cell) and NUP153 (6-10 top cell).

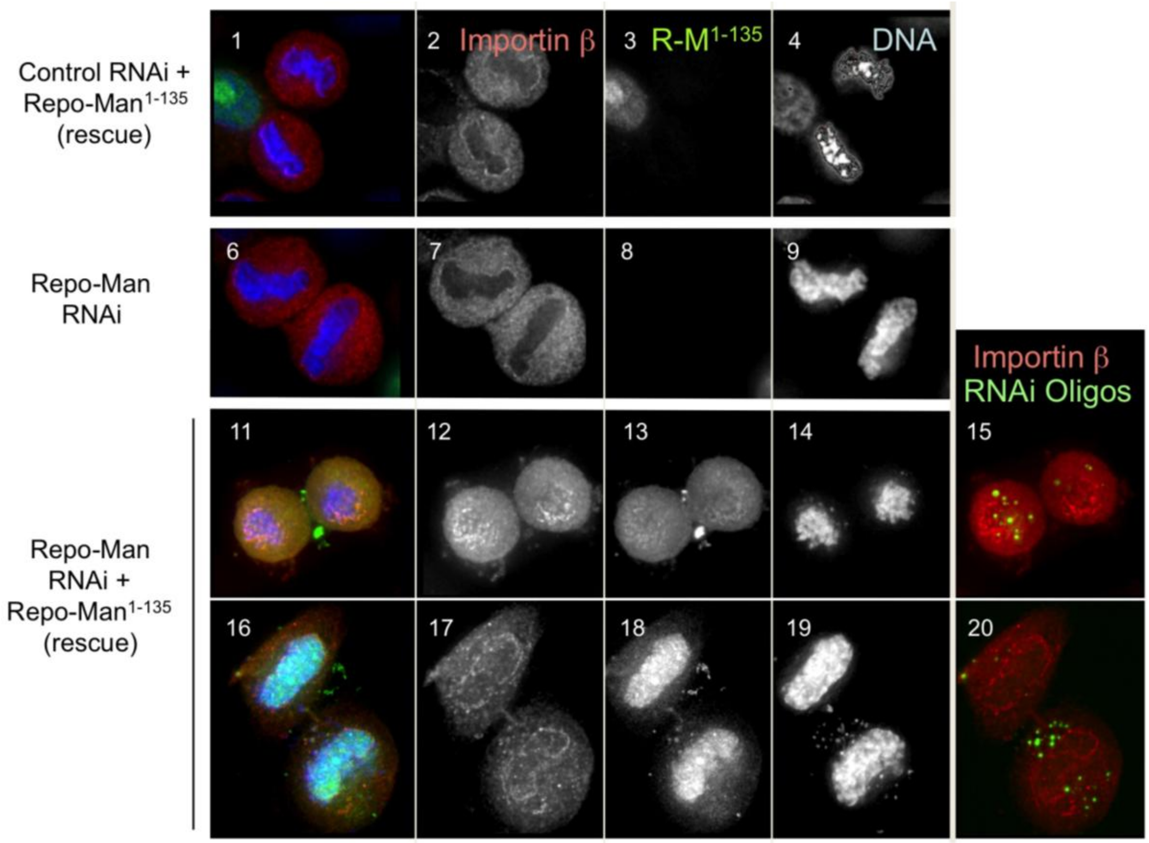

Figure 7. Expression of Repo-Man$^{1\text{-}135}$ Restores Proper Importin β Accumulation around Chromosomes during Mitotic Exit in a Repo-Man RNAi Background

Importin β localization following control (1-4) or Repo-Man (6-9) RNAi. Panels (11-20), Importin β localization in HeLa cells after Repo-Man RNAi in presence of the rescue construct GFP:Repo-Man$^{1\text{-}135}$.

Supplemental Experimental Procedures

Repo-Man Sequence Alignments

The alignment was produced with T-Coffee [PMID: 10964570] using default parameters, slightly refined manually and viewed with Belvu (Sonnhammer and Hollich, 2005). The amino acid coloring scheme indicates average BLOSUM62 scores (which are correlated with amino acid conservation) for each alignment column: Red (greater than 3),violet (between 3 and 1.5) and yellow (between 1.5 and 0.5). Mutants discussed in the text are labeled in blue. Sequences were obtained from UniProt, ENSEMBL and NCBI-GenBank but were supplemented by manually assembled ESTs and FGENESH+ predicted gene models (Solovyev et al., 2006). Sequences are named according to:
h (human), NCBI:NP_689775, *Homo sapiens*; d (dog), NCBI:XP_534571, *Canis lupus familiaris*; m (mouse), UniProt:Q14B71, *Mus musculus*; o (opossum), ENSEMBL genome & FGENESH, *Monodelphis domestica*; c (chicken), NCBI:XP_417676 & ESTs, *Gallus gallus*;
X (Xenopus), NCBI:ACR33033, *Xenopus laevis*; z (zebrafish), UniProt:A2CEF0, *Danio rerio*; and, p (pufferfish), ENSEMBL genome & FGENESH, *Tetraodon nigroviridis*.

Table S1. Constructs Generated

CONSTRUCT	VECTOR
pTrAP:Repo-Man^{wt}	C1-pTrAP
pTrAP:Repo-Man^{RAXA}	C1-pTrAP
GFP:Repo-Man^{T412A}	p-EGFP N1
GFP:Repo-Man^{T34AT412AT419A} (TA3)	p-EGFP N1
pTrAP:Repo-Man^{TA3}	C1-pTrAP
GFP:Repo-Man^{TA3RAXA}	p-EGFP N1
GFP:Laci-Repo-Man^{wt}	p-EGFP N1
GFP:Repo-Man$^{1-135}$	p-EGFP N1
GFP:Repo-Man$^{1-349}$	p-EGFP N1
GFP:Repo-Man$^{403-1203}$	p-EGFP N1
GST-Repo-Man$^{1-135}$	pGEX4.1
mRFP:gGPP1γ	p-mRFP N1
mRFP:gGPP1β	p-mRFP N1
RanQ69L	p-TRACER_SV40
GFP:Laci-Repo-Man$^{1-135}$	p-EGFP N1
mRFP:Repo-Man$^{1-349}$	p-mRFP N1
GST-Repo-Man$^{161-659}$	pGEX4.1

Table S2. Antibodies Used

Antibody	Source	Dilution
anti-hRepo-Man	Rabbit polyclonal Generated against GST-Repo-Man$^{161-659}$	1:300
anti-α tubulin	SIGMA	1:1000
anti-S tag	mAb gift of Scott Kaufmann, Mayo Clinic, Rochester, MN	1:500
anti-H3Ser10ph	Upstate Biotechnology	1:200
anti-H3Ser28ph	Abcam	1:500
anti-H3Thr3ph	Abcam	1:2000
anti-Aurora Aph	gift of P.A. Eyers, Sheffield	1:500
anti-Importin β	3E9, Abcam	1:1000
anti-Nup153	QE5, Abcam	1:300
mAB414	Covance	1:1000
anti-hLaminA/C	gift of E Shirmer, Edinburgh	1:500
anti-HP1-α	Millipore	1:500
anti-MEL-28	gift of M. Platani, Edinburgh	1:200
anti-Ran	BD	1:500
anti–INCENP	(Mackay and Earnshaw, 1993)	1:500
anti–Lamin B1	Zymed Paisley, UK	1:100
