# Supplementary figures and images for: Repo-Man Coordinates Chromosomal Reorganization with Nuclear Envelope Reassembly during Mitotic Exit
Source: Dev Cell. 2011 Aug 16;21-248(2):328–42. doi: 10.1016/j.devcel.2011.06.020 (PMC3480639; doi:10.1016/j.devcel.2011.06.020)

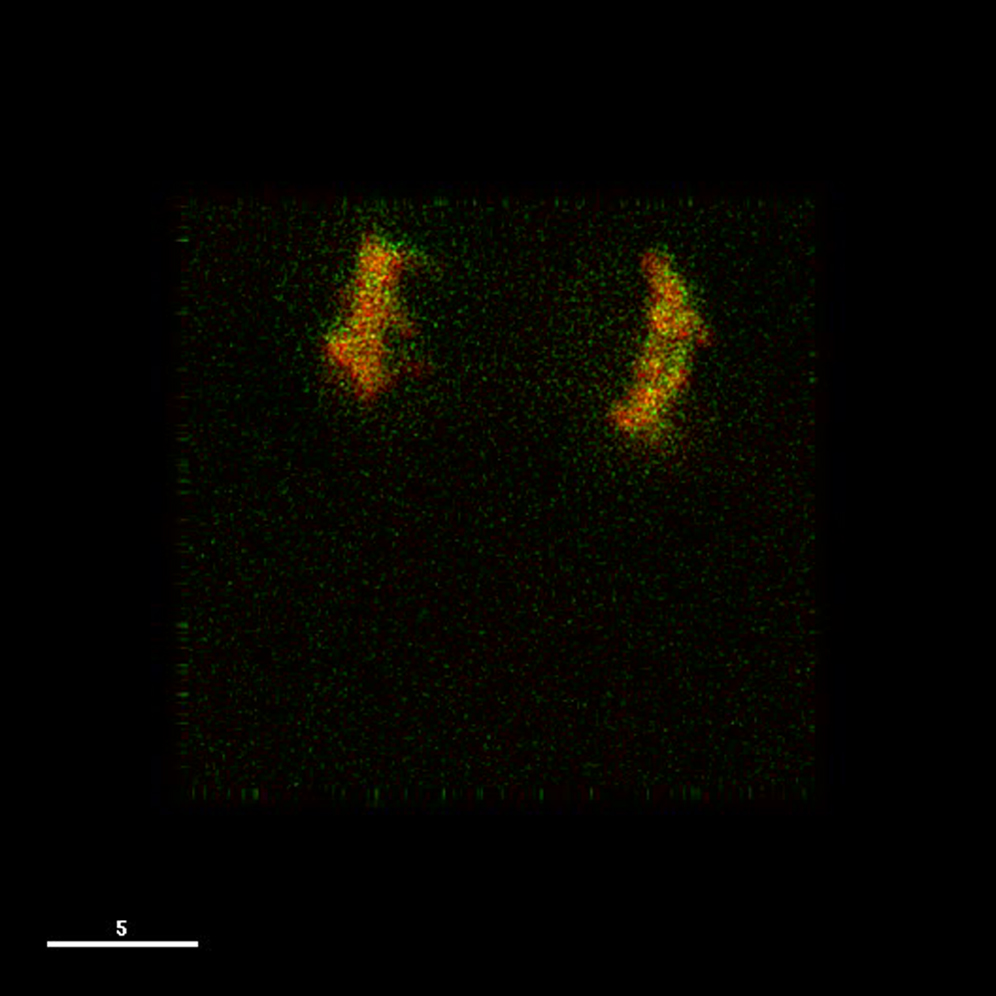

Supplement: Movie S1. GFP:Repo-Man 403–1023 Localizes to Bulk Chromatin after Anaphase Onset — Dt40 cells transiently transfected with GFP:Repo-Man403–1023 and H2B:mRFP were imaged every minute from prometaphase to telophase-cytokinesis. [file mmc2.jpg]

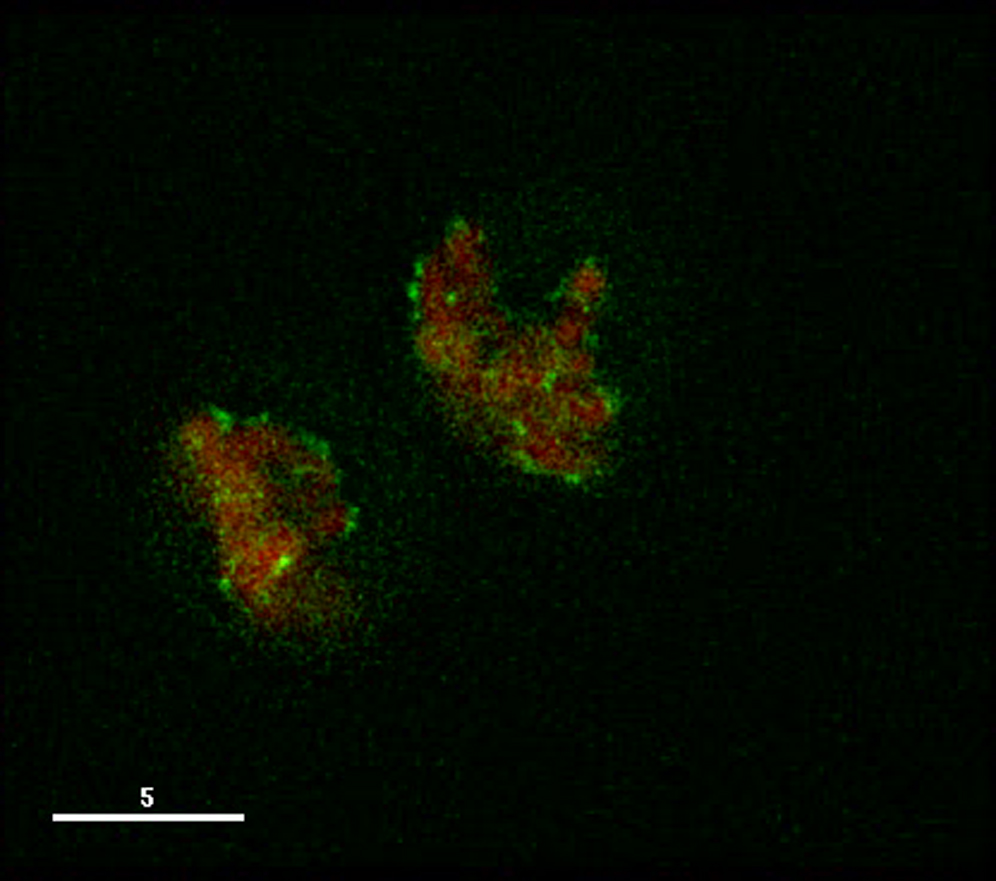

Supplement: Movie S2. GFP:Repo-Man1–397 Localizes to the Chromosomes Periphery in Telophase — Dt40 cells transiently transfected with GFP:Repo-Man403–1023 and H2B:mRFP were imaged every minute from prometaphase to telophase-cytokinesis. [file mmc3.jpg]
